# Psychometric evaluation of the Chinese revised Sensory Integration and Praxis Tests in children with amblyopia

**DOI:** 10.7717/peerj.21431

**Published:** 2026-06-18

**Authors:** Meng Ru, Lu Pan, Yuxing Huang, Wuqiang Luo, Lili Li, Yan Luo, Enwei Lin, Min Kong, Qi Chen, Yali Luo, Hairun Liu, Siyan Huang, Jie Li, Jin Zeng, Yihong Xie, Xin Xiao

**Affiliations:** 1 School of Public Health, Guangxi Medical University, Nanning, China; 2 Visual Science and Optometry Center, The People’s Hospital of Guangxi Zhuang Autonomous Region, Nanning, China; 3 School of Public Health, Guilin Medical University, Guilin, China; 4 School of Public Health and Management, Guangxi University of Chinese Medicine, Nanning, China; 5 Cognitive Sleep Center, The People’s Hospital of Guangxi Zhuang Autonomous Region, Nanning, China; 6 Department of Children’s Rehabilitation Therapy, People’s Hospital of Guangxi Zhuang Autonomous Region, Nanning, China; 7 Department of Ophthalmology, Guangdong Provincial People’s Hospital (Guangdong Academy of Medical Sciences), Southern Medical University, Guangzhou, China; 8 Guangxi Key Laboratory of Eye Health, the People’s Hospital of Guangxi Zhuang Autonomous Region, Nanning, China; 9 Department of Scientific Research, The People’s Hospital of Guangxi Zhuang Autonomous Region, Nanning, China

**Keywords:** Sensory integration, Amblyopia, Sensory integration and praxis tests, Reliability, Validation

## Abstract

**Background:**

Children with amblyopia commonly present with sensory integration difficulties, however, there is a paucity of validated tools to assess these issues in this clinical population. This study aimed to evaluate the psychometric properties of the Chinese Revised version of the Sensory Integration and Praxis Tests (SIPT-R) for use in children with amblyopia.

**Methods:**

We conducted a psychometric validation study using baseline data from the China Amblyopia Behavioral Cohort (CABC). Participants included 629 children aged 4–9 years diagnosed with amblyopia; subtypes included bilateral ametropic (55.7%), anisometropic (38.8%), and strabismic amblyopia (5.6%). Based on severity, 32.1% were classified as mild amblyopia, 59.3% as moderate amblyopia, and 8.6% as severe. Because the learning ability subscale was only applicable to children aged 6 years and older, scale structure differed between age groups. Thus, reliability and validity were evaluated separately for the aged 4–5 and 6–9 years. Internal consistency and construct validity were examined *via* Cronbach’s α, split-half reliability, and confirmatory factor analysis (CFA). Convergent and discriminant validity were assessed *via* factor loadings, composite reliability (CR), and average variance extracted (AVE).

**Results:**

The SIPT-R demonstrated excellent internal consistency (Cronbach’s α = 0.968 − 0.969) and strong subscale reliability (α = 0.906 − 0.959). All split-half coefficients exceeded 0.80. CFA indicated an acceptable model fit for both age groups: for aged 4–5 years, *χ*^2^/*df* = 2.712, root mean square error of approximation (RMSEA) = 0.079, comparative fit index (CFI) = 0.832, incremental fit index (IFI) = 0.833; for aged 6–9 years, *χ*^2^/*df* = 2.951, RMSEA = 0.074, CFI = 0.804, IFI = 0.805. Most items demonstrated adequate factor loadings, with CR values ranging from 0.91 to 0.96 and AVEs between 0.41 and 0.68. Discriminant validity was largely satisfactory; however, a high correlation was observed between tactile defensiveness and proprioception (*r* = 0.86), which exceeded the square root of their respective AVE (0.69), suggesting some overlap between these constructs.

**Conclusions:**

The Chinese revised SIPT-R demonstrates strong reliability and acceptable validity for screening sensory integration function in children with amblyopia. Further research should focus on refining specific subscales and evaluating additional psychometric properties, such as test–retest reliability and criterion validity.

## Introduction

Sensory integration (SI) is a fundamental neurodevelopmental process wherein the brain organizes and interprets multisensory inputs—including visual, auditory, tactile, vestibular, and proprioceptive signals—to produce adaptive behavioural responses. This process underpins higher-order cognitive functions such as attention, memory, and language development (Camarata, Miller & Wallace, 2020; Lane et al., 2019). Impairments in SI, often referred to as sensory integration disorder (SID), are common in children, with an estimated prevalence of 21.9% (Jorquera-Cabrera et al., 2017; Zhao et al., 2021). Children with SID frequently experience marked difficulties in processing and integrating sensory information, which can adversely affect motor coordination, learning, and daily functioning.

Amblyopia, a neurodevelopmental disorder of vision, is a leading causes of visual impairment worldwide, with an estimated prevalence of approximately 1.36% (Chaturvedi, Jamil & Sharma, 2023; Hu et al., 2022). Beyond core visual deficits, growing evidence indicates that amblyopia can disrupt multisensory integration. Specifically, visual dysfunction in these children may interfere with the normal processing of vestibular, proprioceptive, and tactile inputs (Wang et al., 2023), potentially contributing to secondary challenges in motor coordination, attention, and cognitive performance (Webber, 2018; Wong, 2023). Accordingly, children with amblyopia show a higher prevalence of co-occurring SID symptoms (Kim et al., 2022; Su et al., 2019), highlighting the need for reliable, population-specific assessment tools.

The assessment of SI has traditionally relied on instruments such as the Ayres Sensory Integration and Praxis Tests (SIPT) (Dunn, 1991), considered a benchmark for the evaluating praxis. However, the SIPT is normed on North American children, and its use is limited by substantial training requirements, cost, and interpretive complexity (Mailloux et al., 2023). Other available tools, including the Sensory Processing Measure-2 (SPM-2) (Narzisi et al., 2022) and the Evaluation in Ayres Sensory Integration^®^ (EASI) (Mailloux et al., 2021; Mailloux et al., 2017; Schaaf et al., 2023), also present constraints such as lengthy administration and operational complexity, rendering them more suitable for in-depth clinical evaluation than for large-scale screening. Therefore, a clear need exists for psychometrically sound, efficient, and accessible measures capable of accurately capturing SI profiles in specific clinical groups, including children with amblyopia.

To address this gap, the Chinese Revised Version of the Sensory Integration and Praxis Tests (SIPT-R) was developed by Professor Guiying Ren’s team (Ren et al., 1994; see Supplementary Text 4). This 58-item instrument has shown strong reliability and validity in typically developing Chinese children and is now widely used nationwide. Nevertheless, its applicability in pediatric populations with visual impairments such as amblyopia remains formally unexamined. Children with amblyopia often develop altered sensory-processing strategies—for instance, relying more heavily on proprioceptive and vestibular cues to compensate for visual deficits (Niechwiej-Szwedo, Colpa & Wong, 2019)—which may affect how the tool performs in this group. Without population-specific validation, the clinical utility of the SIPT-R for identifying SI abnormalities in amblyopic children remains uncertain.

Using baseline data from the China Amblyopia Behavioral Cohort (CABC), the present study aimed to systematically evaluate the psychometric properties of the SIPT-R in children aged 4–9 years with amblyopia. We examined internal consistency, structural validity, convergent validity, and discriminant validity. Our objective was to establish the SIPT-R as a standardized, validated instrument for assessing sensory integration in this clinical population, thereby supporting early identification and intervention planning for co-occurring SID.

## Methods

### Study design and participants

This psychometric validation study drew on baseline data from the CABC, a prospective multicenter study. The sample comprised 629 children aged 4–9 years with a confirmed diagnosis of amblyopia, recruited from participating centers in Guangxi, Guangdong, and Sichuan Provinces between April 2024 and June 2025. The protocol was approved by the Institutional Review Board of the Guangxi Zhuang Autonomous Region People’s Hospital (Approval No. KY-KJT-2023-285) and conducted in accordance with the Declaration of Helsinki. Written informed consent was obtained from the parents or guardians of each participant.

Amblyopia was diagnosed according to the criteria established by the Ophthalmology Branch of the Chinese Medical Association (Chinese Association for Pediatric Ophthalmology and Strabismus, Pediatric Ophthalmology and Strabismus Group of Chinese Ophthalmologist Association & Wei, 2021). Inclusion criteria were: (1) diagnosis of anisometropic amblyopia (interocular spherical equivalent difference ≥ 1.5D or cylindrical difference ≥ 1.0D), bilateral ametropic amblyopia (caused by bilateral refractive errors, with binocular equivalent spherical anisometropia <0.75D), or strabismic amblyopia; (2) best-corrected visual acuity (BCVA) in the amblyopic eye ≥ 0.1 logMAR, with severity classified as mild (BCVA >0.09 to <0.30 logMAR), moderate (BCVA ≥ 0.30 to <0.70 LogMAR), or severe (BCVA ≥ 0.70 LogMAR) (Ghasia & Tychsen, 2024); (3) absence of other ocular or systemic conditions that could affect sensory or neurological function; and (4) no history of ocular surgery. Children unable to cooperate with testing or with other conditions potentially affecting central nervous system development were excluded.

### Data collection and quality control

Data were collected using a structured electronic questionnaire administered *via* the Wenjuanxing platform. Parents completed the instrument with direct assistance from trained research staff to ensure understanding and accuracy. A multistage quality-control protocol was implemented: (1) real-time clarification of ambiguous responses during administration; (2) automated checks by the platform for missing or implausible values; and (3) an independent review by two researchers to verify completeness, logical consistency, and adherence to the inclusion criteria. Questionnaires failing any of these stages were excluded. Of 645 distributed questionnaires, 629 were retained for analysis, yielding a response rate of 97.5%.

The final sample consisted of 275 children aged 4–5 years and 354 aged 6–9 years. The sample size for both age groups exceeded the recommended minimum of 5–10 participants per item for stable factor analysis (Yong & Pearce, 2013), given the 47-item (ages 4–5) and 55-item (ages 6–9) versions of the SIPT-R used.

### Measures and Instruments

#### Demographic and clinical data

Demographic variables (age, gender, ethnicity, birth weight, maternal smoking/alcohol history, family history of myopia/amblyopia) were collected *via* parent reports.

Ophthalmic examinations were performed on the same day as SIPT-R administration. These included measurement of BCVA using an E-letter standard logarithmic visual acuity chart under standardized conditions, fundus examination, and assessment of ocular alignment. To ensure accurate refraction, cycloplegia was induced with 1% cyclopentolate hydrochloride eye drops before retinoscopy. All procedures were carried out by certified optometrists following established pediatric protocols.

#### SIPT-R

The SIPT-R is a parent-reported questionnaire adapted for the Chinese children. It assesses sensory integration across five domains: vestibular function (14 items), tactile defensiveness (21 items), proprioception (12 items), learning ability (eight items, for children aged ≥6 years), and specific issues (three items, for children aged ≥10 years). Children aged 4–5 years therefore completed a 47-item version (excluding learning ability and specific issues), while those aged 6–9 years completed a 55-item version (excluding specific issues). Items are rated on a 5-point Likert scale from 1 (“Never”) to 5 (“Always”). Raw scores are converted to age-normal T-scores (mean = 50, SD = 10) for interpretation.

### Statistical analysis

Analysis were conducted using IBM SPSS Statistics 27.0 and AMOS 28.0. Continuous variables were assessed for normally with the Kolmogorov–Smirnov test; non-normally distributed variables are summarized as medians and interquartile ranges. Categorical variables are presented as counts and percentages.

#### Item analysis

Spearman’s correlation was used to examine the relationship between each item and its hypothesized factor. A correlation coefficient ≥ 0.40 was considered acceptable (Cella et al., 2022).

#### Reliability

Internal consistency was evaluated with Cronbach’s alpha (α ≥ 0.70 considered acceptable; Strömbeck et al., 2025) and split-half reliability (Spearman-Brown coefficient). The unequal-length Spearman-Brown formula was applied for the 21-item tactile defensiveness subscale.

#### Validity

Structural validity: Confirmatory factor analysis (CFA) was used to test the predefined factor structure. Given non-normal data, robust parameter estimates were obtained *via* a nonparametric bootstrap procedure with 5,000 resamples. Model fit was judged against the following criteria: *χ*^2^*/df* < 3.0, root-mean-square error of approximation (RMSEA) < 0.08, and comparative fit index (CFI)/incremental fit index (IFI) > 0.80.

Convergent validity: This was assessed *via* factor loadings (acceptable > 0.50), composite reliability (CR > 0.70), and average variance extracted (AVE > 0.36) (Strömbeck et al., 2025; Vicente, 2023).

Discriminant validity: This was considered adequate if the square root of a factor’s AVE exceeded its correlations with all other factors.

## Results

### Participant characteristics

The study included 629 children diagnosed with amblyopia, of whom 275 (43.7%) were aged 4–5 years and 354 (56.3%) aged 6–9 years. Demographic and clinical characteristics are summarized in Table 1. Most participants were Han ethnicity, and no maternal smoking or alcohol use during pregnancy was reported. A family history of myopia was more common than a family history of amblyopia. The majority of children had normal birth weights (2,500–4,000 g). Regarding visual function, the median BCVA in the better eye was 0.2 logMAR (interquartile range (IQR): 0.2, 0.4) in children aged 4–5 years and 0.1 logMAR (IQR: 0, 0.2) in children aged 6–9 years. Moderate amblyopia was the most common severity, especially among younger children (72.7%), and bilateral ametropic amblyopia was the predominant type overall. Scores on the SIPT-R indicated greater sensory-integration challenges in younger children across the vestibular, tactile defensiveness, and proprioception domains (Table 1).

**Table 1:** Demographics, clinical characteristics, and SIPT-R scores of children aged 4–9 years. Data are presented as number (%) for categorical variables and median (P_25_, P_75_) for continuous variables. BCVA refers to best-corrected visual acuity. SIPT-R scores indicate sensory integration performance in vestibular function, tactile defensiveness, proprioception, and learning ability.

Variable	4–5 years (*N* = 275)	6–9 years (*N* = 354)
Gender (N (%))		
Boy	143 (52.0)	181 (51.1)
Girl	132 (48.0)	173 (48.9)
Ethnicity		
Han	184 (66.9)	229 (64.7)
Ethnic minorities	91 (33.1)	125 (35.3)
Maternal smoking history (N (%))		
Yes	5 (1.8)	6 (1.7)
No	270 (98.2)	348 (98.0)
Maternal alcohol use history (N (%))		
Yes	26 (9.5)	30 (8.5)
No	249 (90.5)	324 (91.2)
Family history of myopia (N (%))		
Yes	63 (22.9)	61 (17.2)
No	212 (77.1)	293 (82.5)
Family history of amblyopia (N (%))		
Yes	18 (6.5)	25 (7.1)
No	257 (93.5)	329 (92.7)
Child’s birth weight (N (%))		
≤ 2,500 g	34 (12.4)	54 (15.3)
2,500–4,000 g	227 (82.5)	283 (79.7)
≥ 4,000 g	14 (5.1)	17 (4.8)
BCVA (Median (P_25_, P_75_))		
Better eye	0.2 (0.2, 0.4)	0.1 (0, 0.2)
Worse eye	0.4 (0.3, 0.5)	0.3 (0.2, 0.4)
Severity of amblyopia (N (%))		
Mild	46 (16.7)	156 (44.1)
Moderate	200 (72.7)	173 (48.9)
Severe	29 (10.5)	25 (7.1)
Types of amblyopia (N (%))		
Bilateral ametropic amblyopia	167 (60.7)	183 (51.7)
Anisometropic amblyopia	97 (35.3)	147 (41.5)
Strabismic amblyopia	11 (4.0)	24 (6.8)
SIPT-R Scores (Median (P_25_, P_75_))		
Vestibular Function	59 (51, 68)	56 (46, 64)
Tactile Defensiveness	60 (50, 70)	57 (47, 63)
Proprioception	60 (51, 65)	58 (47, 64)
Learning Ability	–	54 (46, 62)

**Notes.**

Severity of amblyopia was classified based on the BCVA of the amblyopic eye (AE): Mild: >0.09 and <0.30 LogMAR; Moderate: ≥0.30 and <0.70 LogMAR; Severe: ≥0.70 LogMAR.

### Item analysis

Strong item–total correlations were observed in both age groups, supporting the internal consistency of the SIPT-R. In children aged 4–5 years, correlations between each subscale and the total score ranged from 0.84 to 0.89 (*p* < 0.01). Inter-factor correlations were moderate (*r* = 0.60–0.74, *p* < 0.01), indicating distinguishable sub-constructs. Similarly, in the aged 6–9 years, subscale-total correlations ranged from 0.84 to 0.92 (*p* < 0.01), with inter-factor correlations between 0.60 and 0.78 (*p* < 0.01). These results suggest that each subscale contributed meaningfully to the total score while retaining adequate distinctiveness (see Table 2 for details).

**Table 2:** Spearman correlations among SIPT-R subscales and total scores in children aged 4–9 years. Spearman correlation coefficients are shown for interfactor and factor-total relationships. Data are presented separately for children aged 4–5 years and 6–9 years. *P* < 0.01 indicates statistical significance. SIPT-R refers to the total sensory integration score, and subscales include vestibular function, tactile defensiveness, proprioception, and learning ability.

Subscales		1	2	3	4	5
4–5 years						
1	SIPT-R	1				
2	Vestibular Function	0.84^**^	1			
3	Tactile Defensiveness	0.89^**^	0.63^**^	1		
4	Proprioception	0.87^**^	0.60^**^	0.74^**^	1	
6–9 years						
1	SIPT-R	1				
2	Vestibular Function	0.85^**^	1			
3	Tactile Defensiveness	0.92^**^	0.70^**^	1		
4	Proprioception	0.85^**^	0.60^**^	0.78^**^	1	
5	Learning Ability	0.84^**^	0.65^**^	0.67^**^	0.75^**^	1

**Notes.**

** *p* < 0.01.

### Reliability

The SIPT-R showed excellent internal consistency in both age groups. Cronbach’s α for the total scale was 0.969 in the aged 4–5 years and 0.968 in the aged 6–9 years. Subscale α coefficients ranged from 0.911 to 0.969 in the younger group and from 0.906 to 0.968 in the older group, all exceeding the 0.70 acceptability threshold. Split-half reliability coefficients were also high, with all values above 0.80, further supporting the scale’s reliability (Table 3).

### Validity

#### Construct validity

**Figure 1:**
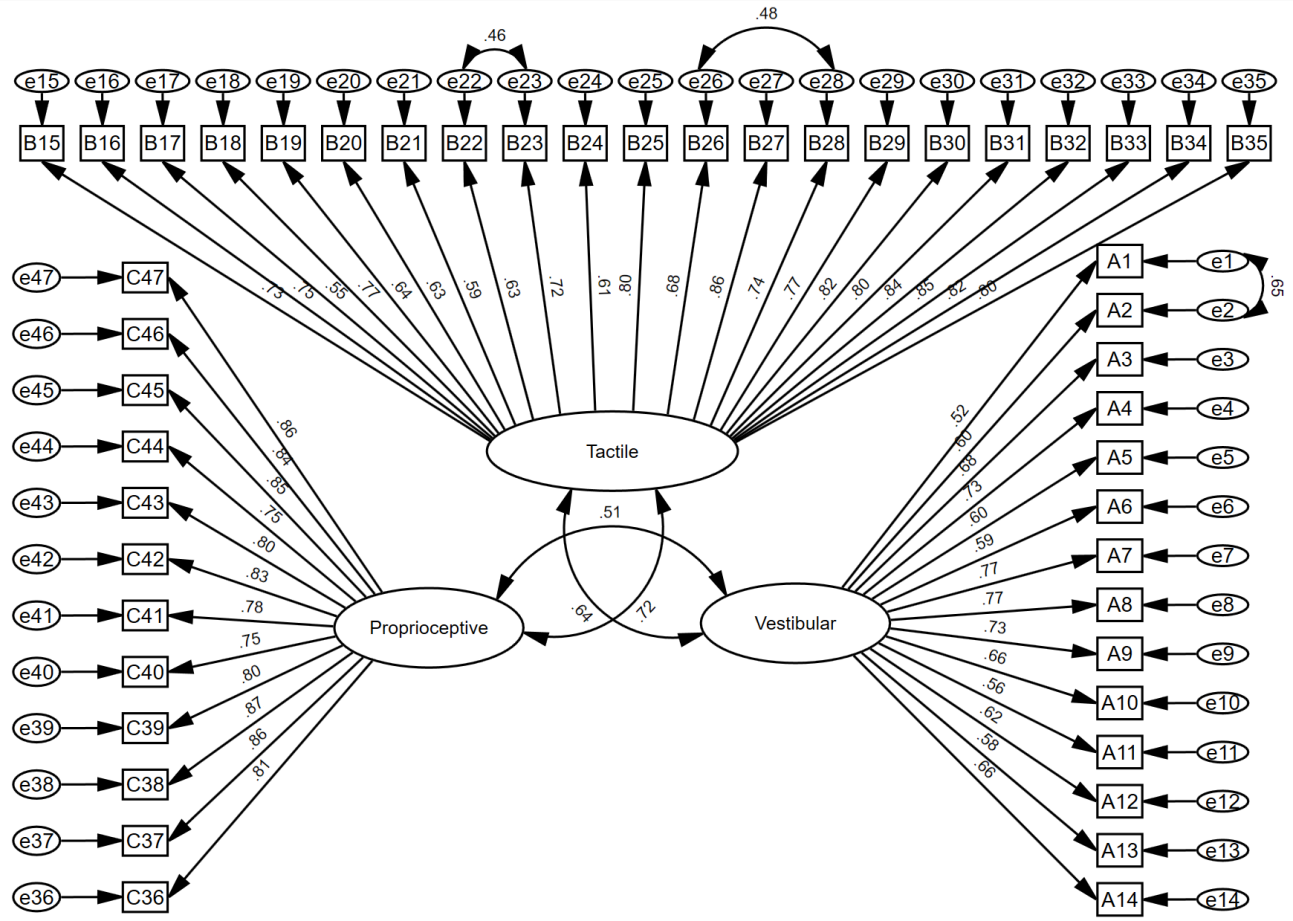
The fitting figure of the modification three-factor model (4–5 years group). Path diagram of the three-factor model representing vestibular function, tactile defensiveness, and proprioception. Rectangles indicate observed variables, ovals indicate latent factors, and single-headed arrows show factor loadings.

**Figure 2:**
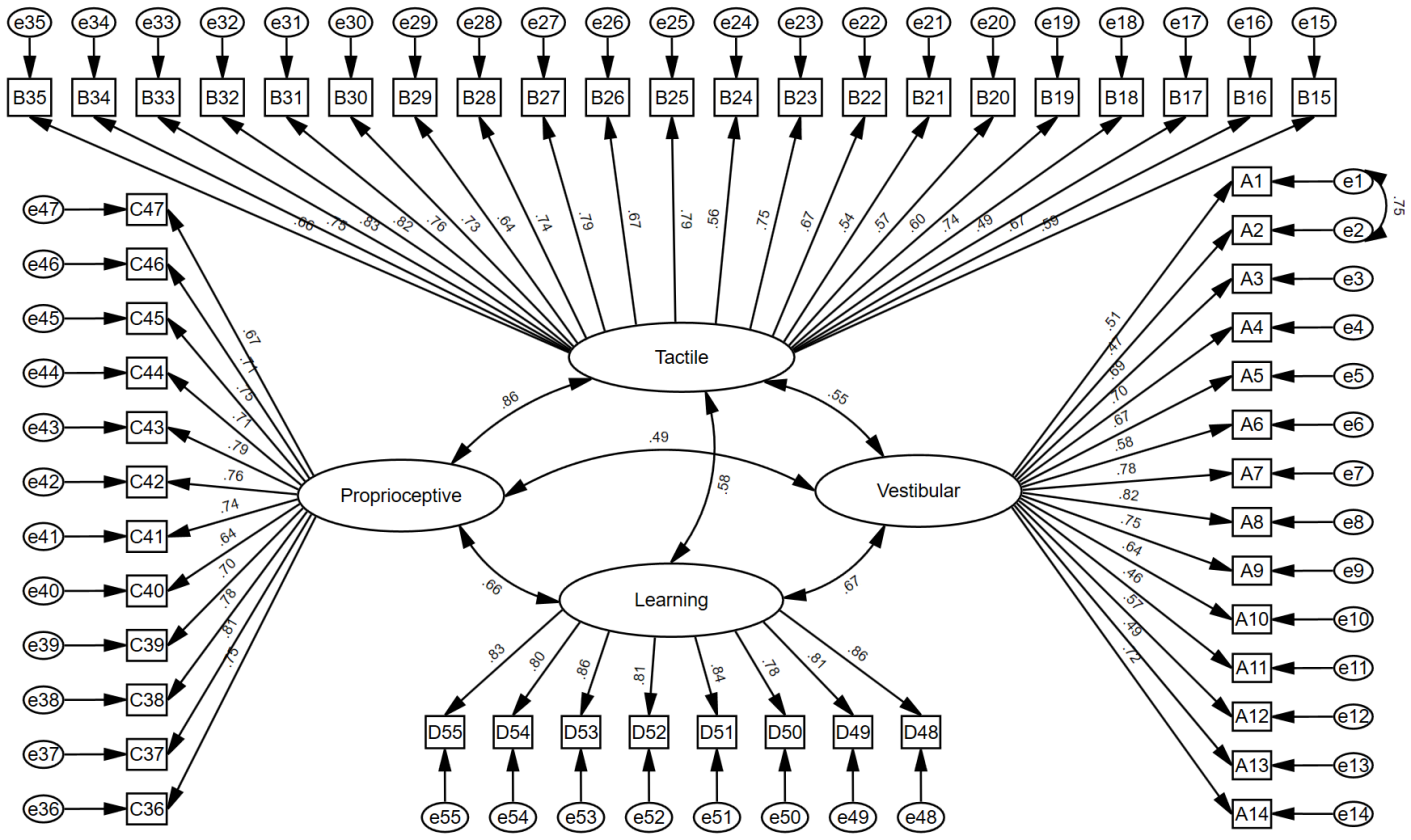
The fitting figure of the modification four-factor model (6–9 years group). Path diagram of the four-factor model including vestibular function, tactile defensiveness, proprioception, and learning ability. Rectangles indicate observed variables, ovals indicate latent factors, and single-headed arrows show standardized factor loadings for each relationship.

Confirmatory factor analysis was conducted to evaluate the structural validity of the SIPT-R. For aged 4–5 years, a three-factor model (vestibular function, tactile defensiveness, and proprioception) was tested. The initial model fit was suboptimal; after incorporating three theoretically justified residual correlations (e1–e2, e22–e23, e26–e28) based on modification indices and item-content similarity, fit indices improved to acceptable levels: *χ^2^/df* = 2.712, RMSEA = 0.079, CFI = 0.832, and IFI = 0.833 (Fig. 1). For aged 6–9 years, a four-factor model (adding learning ability) was tested. After correlating the residuals of items e1 and e2, the model fit improved to *χ^2^/df* = 2.951, RMSEA = 0.074, CFI = 0.804, and IFI = 0.805 (Fig. 2), indicating an acceptable model-data fit (Table 4).

**Table 3:** Reliability coefficients of the SIPT-R in children with amblyopia aged 4–9 years. Cronbach’s α and split-half reliability (Spearman–Brown) for the SIPT-R total scale and subscales among children aged 4–5 years and 6–9 years. All subscales demonstrated high internal consistency.

Subscales	Items	4–5 years		6–9 years	
		Cronbach’ s α	Spearman-Brown	Cronbach’ s α	Spearman-Brown
SIPT-R	47/55	0.969	0.856	0.968	0.813
Vestibular Function	14	0.911	0.883	0.906	0.876
Tactile Defensiveness	21	0.959	0.914	0.943	0.888
Proprioception	12	0.959	0.954	0.930	0.940
Learning Ability	8	–	–	0.944	0.913

#### Convergent validity

Convergent validity was satisfactory in both models. In the three-factor model, all factor loadings exceeded 0.50. In the four-factor model, two items (A2, A11) had marginally lower loadings (0.46–0.47); all other items loaded strongly on their respective factors (>0.50).

Composite reliability (CR) values ranged from 0.91 to 0.96, and average variance extracted (AVE) values ranged from 0.41 to 0.68, meeting recommended thresholds and supporting the convergent validity of the subscales (Table 5).

**Table 4:** Model fit indices for confirmatory factor analysis of the SIPT-R. Fit indices (*χ^2^/df*, CFI, IFI, RMSEA) for three- and four-factor CFA models and their adjusted versions. Acceptable thresholds: *χ^2^/df* < 3, CFI and IFI < 0.80, RMSEA < 0.08. Results indicate that the adjusted models provided improved fit.

Model	*χ* ^2^ */df*	CFI	IFI	RMSEA
Three-factor model	2.970	0.806	0.807	0.085
Four-factor model	3.152	0.784	0.785	0.078
Adjust Three-factor model	2.712	0.832	0.833	0.079
Adjust Four-factor model	2.951	0.804	0.805	0.074
Evaluation Criteria	<3.000	>0.800	>0.800	<0.080

**Table 5:** Standardized regression coefficients and validity indices of the SIPT-R. Standardized factor loadings of SIPT-R items across subscales for children aged 4–5 years and 6–9 years. Composite reliability (CR) and average variance extracted (AVE) values are reported for each construct. Most items showed factor loadings >0.50, supporting convergent validity. *** *P* < 0.001 indicates that the factor loadings of these SIPT-R scale items are all highly statistically significant (rejecting the hypothesis that the coefficient is 0), proving that the structural validity of the scale is very robust.

		4–5 years				6–9 years			
		Estimate	*p*	CR	AVE	Estimate	*p*	CR	AVE
Vestibular Function	A1	0.52	–	0.91	0.43		0.51	–	0.91	0.41
	A2	0.60	^***^			0.47	^***^		
	A3	0.68	^***^			0.69	^***^		
	A4	0.73	^***^			0.70	^***^		
	A5	0.60	^***^			0.67	^***^		
	A6	0.59	^***^			0.58	^***^		
	A7	0.77	^***^			0.78	^***^		
	A8	0.77	^***^			0.82	^***^		
	A9	0.73	^***^			0.75	^***^		
	A10	0.66	^***^			0.64	^***^		
	A11	0.56	^***^			0.46	^***^		
	A12	0.62	^***^			0.57	^***^		
	A13	0.59	^***^			0.50	^***^		
	A14	0.66	^***^			0.72	^***^		
Tactile Defensiveness	B15	0.73	–	0.96	0.55	0.59	–	0.95	0.48
	B16	0.75	^***^			0.68	^***^		
	B17	0.55	^***^			0.50	^***^		
	B18	0.77	^***^			0.74	^***^		
	B19	0.64	^***^			0.60	^***^		
	B20	0.63	^***^			0.57	^***^		
	B21	0.59	^***^			0.54	^***^		
	B22	0.63	^***^			0.67	^***^		
	B23	0.72	^***^			0.75	^***^		
	B24	0.61	^***^			0.56	^***^		
	B25	0.80	^***^			0.79	^***^		
	B26	0.68	^***^			0.67	^***^		
	B27	0.86	^***^			0.79	^***^		
	B28	0.74	^***^			0.74	^***^		
	B29	0.77	^***^			0.64	^***^		
	B30	0.82	^***^			0.73	^***^		
	B31	0.80	^***^			0.76	^***^		
	B32	0.84	^***^			0.82	^***^		
	B33	0.85	^***^			0.83	^***^		
	B34	0.83	^***^			0.75	^***^		
	B35	0.81	^***^			0.66	^***^		
Proprioception	C36	0.81	–	0.96	0.67	0.75	–	0.93	0.54
	C37	0.86	^***^			0.81	^***^		
	C38	0.88	^***^			0.78	^***^		
	C39	0.80	^***^			0.70	^***^		
	C40	0.76	^***^			0.64	^***^		
	C41	0.78	^***^			0.74	^***^		
	C42	0.83	^***^			0.76	^***^		
	C43	0.80	^***^			0.79	^***^		
	C44	0.75	^***^			0.71	^***^		
	C45	0.85	^***^			0.75	^***^		
	C46	0.84	^***^			0.71	^***^		
	C47	0.86	^***^			0.67	^***^		
Learning Ability	D55	–	–	–	–	0.86	–	0.94	0.68
	D54	–	–			0.84	^***^		
	D53	–	–			0.83	^***^		
	D52	–	–			0.81	^***^		
	D51	–	–			0.86	^***^		
	D50	–	–			0.80	^***^		
	D49	–	–			0.82	^***^		
	D48	–	–			0.78	^***^		

**Notes.**

*** *P* < 0.001.

#### Discriminant validity

Discriminant validity was generally supported, though some overlap was noted. All inter-factor correlations were significant (*p* < 0.01). In the aged 6–9 years, the correlation between tactile defensiveness and proprioception was high (*r* = 0.86), exceeding the square root of the AVE for both factors (0.69), suggesting limited discriminant validity between these two constructs. Correlations among the other factors remained within acceptable limits relative to their respective AVE square roots (Table 6).

**Table 6:** Discriminant validity of SIPT-R subscales in children aged 4–9 years. Diagonal values represent the square root of the average variance extracted (AVE) for each subscale, and off-diagonal values indicate inter-factor correlations. Data are shown separately for children aged 4–5 years and 6–9 years. *** *P* < 0.001 indicates statistically significant correlations. Subscales include vestibular function, tactile defensiveness, proprioception, and learning ability (for 6–9 years only).

Subscales	Vestibular function	Tactile defensiveness	Proprioception	Learning ability
4–5 years				
Vestibular Function	0.43			
Tactile Defensiveness	0.64^***^	0.55		
Proprioception	0.51^***^	0.72^***^	0.67	
Square root of AVE	0.65	0.74	0.82	
6–9 years				
Vestibular Function	0.41			
Tactile Defensiveness	0.55^***^	0.48		
Proprioception	0.49^***^	0.86^***^	0.54	
Learning Ability	0.67^***^	0.58^***^	0.66^***^	0.68
Square root of AVE	0.64	0.69	0.73	0.83

**Notes.**

Diagonal values represent the average variance extracted (AVE) for each factor; off-diagonal values indicate inter-factor correlations.

*** *P* < 0.001.

## Discussion

This study presents the inaugural systematic evaluation of the psychometric properties of the Chinese Revised Version of the Sensory Integration and Praxis Tests (SIPT-R) in children with amblyopia. Using a substantial sample from a multicentre prospective cohort, we found that the SIPT-R demonstrates excellent internal consistency, acceptable structural validity, and generally sound convergent and discriminant validity in children aged 4–9 years. These findings supports the applicability of the SIPT-R as a standardized tool for assessing sensory integration difficulties in this clinical population, particularly in children with mild to moderate amblyopia.

In line with previous research, we observed age-related differences in sensory integration performance. Specifically, children aged 4–5 years showed marginally higher subscale scores than older children. This is consistent with known developmental variations in sensory integration among typically developing children, which reflect the ongoing maturation of multisensory neural circuits in early childhood (Lin et al., 2013). While no studies to date has directly examined sensory integration trajectories in children with amblyopia, our results suggest that their sensory systems follow broadly similar maturational patterns, albeit with potentially delayed or altered integration pathways.

The SIPT-R demonstrated strong reliability, with Cronbach’s α coefficients ranging from 0.906 to 0.969 across all the subscales. These value align with those reported in validation studies of other populations, including typically developing children and those with neurodevelopmental conditions (Schaaf et al., 2023). The slightly higher alpha values in younger children may reflect more uniform behavioural patterns and less reliance on compensatory strategies, whereas older children (aged 6–9 years) may employ more varied, learned coping mechanisms.

Confirmatory factor analysis indicated acceptable structural validity, with improved model fit after incorporating a limited number of theoretically justified residual correlations. The children aged 6–9 years required fewer modifications, suggesting clearer factor differentiation and more stable sensory domains structures with increasing age—a findings consistent with neuroimaging evidence of age-related stabilisation in multisensory pathways (Camarata, Miller & Wallace, 2020).

Convergent validity was satisfactory based on factor loadings, CR, and AVE. However, two items (A2 and A11) in the children aged 6–9 years showed marginally lower loadings, a pattern noted in previous sensory integration scale evaluations (Mailloux et al., 2021). Items assessing subtle tactile or proprioceptive behaviours may be less consistently linked to their latent constructs and could benefit from refinement to improve clarity or sensitivity.

A notable finding was the limited discriminant validity between the Tactile Defensiveness and Proprioception subscales, especially in older children. Previous neurobehavioural research has highlighted overlapping neural networks for tactile and proprioceptive processing, particularly in individuals with atypical sensory development (Sabater-Gárriz et al., 2025). Moreover, children with amblyopia often rely more heavily on tactile and proprioception cues to compensate for visual deficits (Niechwiej-Szwedo et al., 2016; Niechwiej-Szwedo, Colpa & Wong, 2019), which may increase the interdependence between these sensory domains and help explain the observed overlap.

From a clinical perspective, the SIPT-R appears practically useful for capturing sensory integration characteristics relevant to amblyopia. Previous work links atypical sensory integration in amblyopia to visuomotor deficits, impaired spatial awareness, and reduced motor coordination (Wong, 2023). Screening for sensory integration dysfunction may therefore help identify children at risk of broader developmental challenges, including attention difficulties (Kim et al., 2022). Methodologically, this study contributes by validating the SIPT-R in a large, well-characterized clinical sample. Compared to existing tools such as the SIPT, SPM-2, and EASI, the SIPT-R offers shorter administration time, lower cost (Mailloux et al., 2023), and broader population suitability, making it especially appealing for clinical screening and resource-limited settings.

Several strengths of this study should be noted. First, the usd of a substantial multicentre sample from the CABC enhances the generalisability of the findings. Second, comprehensive psychometric analysis support the applicability of the SIPT-R in children with amblyopia. Third, age-stratified analyses provide insights into developmental characteristics and scale performance across age groups, facilitating more targeted assessment and intervention.

Certain limitations should also be acknowledged. First, reliance on parent-reported data may introduce subjective bias, especially for context-dependent or less observable behaviors. Second, test-retest reliability and criterion validity were not assessed due to the lack of a gold-standard measure of sensory integration in this population. Third, the modest sample size for children aged 6–9 years limited evaluation of the “Specific Issues” subscale. Finally, the partial overlap between some subscales suggests that future refinement of the SIPT-R may be warranted, potentially incorporating objective behavioural tasks or neurophysiological measures (*e.g.*, electroencephalogram (EEG) or functional MRI (fMRI)). Despite these limitations, the present findings provide important evidence supporting the use of the SIPT-R in children with amblyopia.

Future research should: (1) conduct longitudinal assessments to evaluate the temporal stability of SIPT-R scores; (2) validate the full SIPT-R, especially in children ≥ 10 years; (3) refine certain subscale items to improve discriminant validity between tactile and proprioceptive domains; and (4) integrate neuroimaging or kinematic data to elucidate the multisensory mechanisms underlying amblyopia.

## Conclusions

In summary, this study provides comprehensive evidence supporting the SIPT-R as a reliable, valid, and clinically useful instrument for assessing sensory integration in children with amblyopia. The tool can assist clinicians and researchers in identifying sensory processing difficulties, guiding individualized intervention strategies, and advancing the understanding of multisensory deficits associated with amblyopia.

##  Supplemental Information

10.7717/peerj.21431/supp-1 Supplemental Information 1 Complete version of the questionnaire administered in the study The file presents all questionnaire items and response options as provided to parents or caregivers during data collection. Including both Chinese and English translation versions

10.7717/peerj.21431/supp-2 Supplemental Information 2 Additional statistical results and supporting analyses complementing the main findings The analyses that pertain specifically to participants aged ¿9 years and to the SIPT-R “Specific Issues” subscale. These analyses were omitted from the main article (which is restricted to children aged 4-9 years) and are provided here for transparency and completeness. All methods and statistical procedures used below follow those described in the main text.

10.7717/peerj.21431/supp-3 Supplemental Information 3 Age-specific conversion of raw questionnaire scores to standardized scores The lookup rules used to transform raw scores into standardized scores across different age groups.

10.7717/peerj.21431/supp-4 Supplemental Information 4 Chinese references cited and their English translations

10.7717/peerj.21431/supp-5 Supplemental Information 5 Variable definitions and coding schemes for all measures included in the dataset Each entry describes a variable name, its meaning, coding values, and scoring direction to facilitate interpretation and reuse of the dataset.

10.7717/peerj.21431/supp-6 Supplemental Information 6 Raw dataset containing demographic characteristics, questionnaire scores, and outcome variables for all participants This dataset includes anonymized participant-level data used for statistical analyses, with each row representing an individual participant and each column corresponding to a measured variable.

## Additional Information and Declarations

### Data Availability

The following information was supplied regarding data availability: The raw measurements are available in the Supplemental Files 3.
