# Supplementary material for: Psychometric evaluation of the Chinese revised Sensory Integration and Praxis Tests in children with amblyopia
Source: PeerJ. 2026 Jun 18;14:e21431. doi: 10.7717/peerj.21431 (PMC13283362; doi:10.7717/peerj.21431)
Supplement: Supplemental Information 1 — The file presents all questionnaire items and response options as provided to parents or caregivers during data collection. Including both Chinese and English translation versions [file peerj-14-21431-s001.docx]

**Supplementary Text 1: English-Chinese translation of the Chinese Revised Version of the Sensory Integration and Praxis Tests (SIPT-R)**

**Manuscript:** Psychometric validation of the Chinese Revised Version of the Sensory Integration and Praxis Tests in Children with Amblyopia

**Authors:** Meng Ru^1, 2*^, Lu Pan^2, 3*^, Yuxing Huang^2^, Wuqiang Luo^2^, Lili Li^2^, Yan Luo^2^, Enwei Lin^2^, Min Kong^2^, Qi Chen^2^, Yali Luo^4^, Hairun Liu^5^, Siyan Huang^5^, Jie Li^6^, Jin Zeng^7^, Yihong Xie^1#^, Xin Xiao^2, 8, 9#^

^1^School of Public Health, Guangxi Medical University, Nanning 530021, Guangxi, China

^2^Visual Science and Optometry Center, the People's Hospital of Guangxi Zhuang Autonomous Region, Nanning 530021, Guangxi, China

^3^School of Public Health, Guilin Medical University, Guilin 541199, Guangxi, China

^4^School of Public Health and Management, Guangxi University of Chinese Medicine, Nanning 530021, Guangxi, China

^5^Cognitive Sleep Center, the People's Hospital of Guangxi Zhuang Autonomous Region, Nanning 530021, Guangxi, China

^6^Department of Children's Rehabilitation Therapy, People's Hospital of Guangxi Zhuang Autonomous Region, Nanning 530021, Guangxi, China

^7^Department of Ophthalmology, Guangdong Provincial People's Hospital (Guangdong Academy of Medical Sciences), Southern Medical University, Guangzhou 510000, Guangdong, China

^8^Department of Scientific Research, the People's Hospital of Guangxi Zhuang Autonomous Region, Nanning 530021, Guangxi, China

^9^Guangxi Key Laboratory of Eye Health, the People's Hospital of Guangxi Zhuang Autonomous Region, Nanning 530021, Guangxi, China

^*^Meng Ru and Lu Pan contributed equally to this study and should be considered co-first authors

**Corresponding Author**:

Xiao Xin^2, 8, 9^

Visual Science and Optometry Center, the People's Hospital of Guangxi Zhuang Autonomous Region, No. 6 Taoyuan Road, Nanning, Guangxi, 530021, China

Email address: [xiaoxi3891@163.com;](mailto:xiaoxi3891@163.com);)

Xie Yihong^1^

School of Public Health, Guangxi Medical University, No. 22 Shuangyong Road, Nanning, Guangxi, 530021, China

Email address: [gxxieyihong@163.com](mailto:gxxieyihong@163.com)

^#^Xiao Xin and Xie Yihong contributed equally to this work and are co-correspondence authors.

**The English translation version of SIPT-R**

| Based on the child's situation, select one option from “Never,” “Rarely,” “Sometimes,” “Often,” or “Always.” If there are multiple options in the question, only one that applies is sufficient. | | Never | Rarely | Sometimes | Of  ten | Always |
| --- | --- | --- | --- | --- | --- | --- |
| **A** | **Vestibular Function** |  |  |  |  |  |
| A1 | Particularly fond of spinning chairs or amusement park rides, without getting dizzy. | 5 | 4 | 3 | 2 | 1 |
| A2 | Enjoys spinning or running in circles without getting dizzy or tired. | 5 | 4 | 3 | 2 | 1 |
| A3 | Even though they can see, they still frequently bump into tables, chairs, other people, pillars, and walls. | 5 | 4 | 3 | 2 | 1 |
| A4 | Poor coordination between both hands when moving, eating, drumming, or drawing, often forgetting the other side. | 5 | 4 | 3 | 2 | 1 |
| A5 | Clumsy and prone to falling, he still appears heavy when being pulled. | 5 | 4 | 3 | 2 | 1 |
| A6 | Lying face down on the floor or bed, unable to raise the head, neck, or chest | 5 | 4 | 3 | 2 | 1 |
| A7 | Climbing up and down, running in and out, ignoring warnings | 5 | 4 | 3 | 2 | 1 |
| A8 | Restless and fidgety, touching and pulling at things, refusing to listen to advice, and unresponsive to punishment. | 5 | 4 | 3 | 2 | 1 |
| A9 | Likes to provoke people, cause trouble, and play pranks. | 5 | 4 | 3 | 2 | 1 |
| A10 | Often talks to oneself, repeats what others say, and likes to recite advertising slogans. | 5 | 4 | 3 | 2 | 1 |
| A11 | On the surface, it appears to be left-handed, but in reality, both hands are used, and there is no fixed hand for use. | 5 | 4 | 3 | 2 | 1 |
| A12 | Can't tell left from right, often wears shoes and clothes backwards | 5 | 4 | 3 | 2 | 1 |
| A13 | When encountering elevators or staircases in unfamiliar places, one may hesitate to use them or move slowly. | 5 | 4 | 3 | 2 | 1 |
| A14 | Poor organizational skills, often messing things up, dislikes tidying up their environment | 5 | 4 | 3 | 2 | 1 |
| **B** | **Tactile Defensiveness** |  |  |  |  |  |
| B15 | Extremely irritable with loved ones, argumentative, and fearful in unfamiliar environments. | 5 | 4 | 3 | 2 | 1 |
| B16 | Being afraid of new situations and often asking to leave shortly after arriving. | 5 | 4 | 3 | 2 | 1 |
| B17 | Picky eating, refusing to eat vegetables or soft foods | 5 | 4 | 3 | 2 | 1 |
| B18 | Shy, anxious, likes solitude, doesn't like playing with others. | 5 | 4 | 3 | 2 | 1 |
| B19 | Easily becomes attached to their mother or a specific person, dislikes unfamiliar environments, likes to be hugged | 5 | 4 | 3 | 2 | 1 |
| B20 | Watching TV or listening to stories, easily moved to tears, shouting or laughing loudly, afraid of scary scenes | 5 | 4 | 3 | 2 | 1 |
| B21 | Severe fear of the dark, dislike of empty houses, always needing someone to accompany them | 5 | 4 | 3 | 2 | 1 |
| B22 | Lazy in bed in the morning, unable to sleep at night, often refusing to go to school, and not wanting to go home after school. | 5 | 4 | 3 | 2 | 1 |
| B23 | Prone to minor illnesses, and after falling ill, they often refuse to go to school without any apparent reason. | 5 | 4 | 3 | 2 | 1 |
| B24 | Often sucks fingers or bites nails, dislikes others helping to cut nails | 5 | 4 | 3 | 2 | 1 |
| B25 | I can't sleep in a different bed, I can't change my blankets or pajamas, and I often worry about sleep issues when I go out. | 5 | 4 | 3 | 2 | 1 |
| B26 | He is very possessive, and often gets angry for no reason when others touch his things. | 5 | 4 | 3 | 2 | 1 |
| B27 | Does not like chatting with others, does not like playing touch games with others, and finds washing his face and taking a bath painful. | 5 | 4 | 3 | 2 | 1 |
| B28 | He is overly protective of his belongings and especially dislikes others approaching him from behind. | 5 | 4 | 3 | 2 | 1 |
| B29 | Afraid of playing with sand and soil, with a tendency toward obsessive-compulsive disorder | 5 | 4 | 3 | 2 | 1 |
| B30 | Does not like direct eye contact and often has to use hand gestures to express his needs. | 5 | 4 | 3 | 2 | 1 |
| B31 | Slow or overly intense response to danger and pain | 5 | 4 | 3 | 2 | 1 |
| B32 | Hearing but not seeing, being overly quiet, having an indifferent expression, and laughing for no reason. | 5 | 4 | 3 | 2 | 1 |
| B33 | Excessive quietness or insistence on strange gameplay | 5 | 4 | 3 | 2 | 1 |
| B34 | Likes to bite people, often bites the same friends, and breaks things for no reason. | 5 | 4 | 3 | 2 | 1 |
| B35 | Introverted, weak, prone to crying, and often touches their genitals. | 5 | 4 | 3 | 2 | 1 |
| **C** | **Proprioceptive** |  |  |  |  |  |
| C36 | Slow and clumsy when putting on and taking off clothes, buttons, zippers, and tying shoelaces. | 5 | 4 | 3 | 2 | 1 |
| C37 | Stubborn, prejudiced, unsociable, reclusive | 5 | 4 | 3 | 2 | 1 |
| C38 | Often dropping rice grains while eating, unable to control saliva | 5 | 4 | 3 | 2 | 1 |
| C39 | Unclear speech, poor pronunciation, slow language development | 5 | 4 | 3 | 2 | 1 |
| C40 | Lazy, slow to act, inefficient | 5 | 4 | 3 | 2 | 1 |
| C41 | Doesn't like doing somersaults, rolling around, or climbing high. | 5 | 4 | 3 | 2 | 1 |
| C42 | Still unable to wash hands, wipe face, cut paper, or wipe bottom independently while attending kindergarten. | 5 | 4 | 3 | 2 | 1 |
| C43 | Still unable to use chopsticks, hold a pen, climb, or swing on a swing in kindergarten (senior and middle classes). | 5 | 4 | 3 | 2 | 1 |
| C44 | Particularly sensitive to minor injuries, overly dependent on others for care. | 5 | 4 | 3 | 2 | 1 |
| C45 | Not good at playing with building blocks, putting things together, lining up, or throwing balls. | 5 | 4 | 3 | 2 | 1 |
| C46 | Afraid of heights, refuses to walk on the balance beam | 5 | 4 | 3 | 2 | 1 |
| C47 | It's easy to get lost in a new and unfamiliar environment. | 5 | 4 | 3 | 2 | 1 |
| **D** | **Learning Ability (For ages 6 and above)** |  |  |  |  |  |
| D48 | Seems to have normal intelligence, but has particular difficulty learning to read or do arithmetic. | 5 | 4 | 3 | 2 | 1 |
| D49 | Often skips words when reading. Often omits characters or lines when copying. Often reverses strokes when writing. | 5 | 4 | 3 | 2 | 1 |
| D50 | Inattentive, unable to sit still, often looking around during class | 5 | 4 | 3 | 2 | 1 |
| D51 | Difficulty coloring with crayons or writing with a pen, slow writing speed, and frequently writing outside the lines. | 5 | 4 | 3 | 2 | 1 |
| D52 | Reading books easily strains my eyes, and I am particularly afraid of mathematics. | 5 | 4 | 3 | 2 | 1 |
| D53 | Although they have good reading skills, they do not understand the meaning of words and are unable to form longer sentences. | 5 | 4 | 3 | 2 | 1 |
| D54 | Special circles in a confusing background that are difficult to see or recognize | 5 | 4 | 3 | 2 | 1 |
| D55 | Unable to effectively fulfill teachers' requirements and complete assignments, often experiencing severe frustration. | 5 | 4 | 3 | 2 | 1 |
| **E** | **Specific Issues (For ages 10 and above)** |  |  |  |  |  |
| E56 | Poor ability to use tools, unable to perform manual labor or household chores well | 5 | 4 | 3 | 2 | 1 |
| E57 | It is difficult to keep one's own desk or surrounding area clean and tidy. | 5 | 4 | 3 | 2 | 1 |
| E58 | Overreacting to situations, unable to control emotions, prone to negativity | 5 | 4 | 3 | 2 | 1 |

**The Chinese translation version of SIPT-R**

| 根据儿童的情况在“从不这样”、“很少这样”、“有时候”、“常常如此”、“总是如此”选择一项。题中若包括多项，只要有一项符合就算。 | | 从不这样 | 很少这样 | 有时候 | 常常如此 | 总是如此 |
| --- | --- | --- | --- | --- | --- | --- |
| **一、** | **前庭功能** |  |  |  |  |  |
| 1 | 特别爱玩旋转的凳椅或游乐设施，而不会晕 | 5 | 4 | 3 | 2 | 1 |
| 2 | 喜欢旋转或绕圈子跑，而不晕不累 | 5 | 4 | 3 | 2 | 1 |
| 3 | 虽看到了仍常碰撞桌椅、旁人、柱子、门墙 | 5 | 4 | 3 | 2 | 1 |
| 4 | 行动、吃饭、敲鼓、画画时双手协调不良，常忘了另一边 | 5 | 4 | 3 | 2 | 1 |
| 5 | 手脚笨拙、容易跌倒，拉他时仍显得笨重 | 5 | 4 | 3 | 2 | 1 |
| 6 | 俯卧地板和床上、头、颈、胸无法抬高 | 5 | 4 | 3 | 2 | 1 |
| 7 | 爬上爬下，跑进跑出、不听劝阻 | 5 | 4 | 3 | 2 | 1 |
| 8 | 不安地乱动，东摸西扯，不听劝阻，处罚无效 | 5 | 4 | 3 | 2 | 1 |
| 9 | 喜欢惹人、捣蛋、恶作剧 | 5 | 4 | 3 | 2 | 1 |
| 10 | 经常自言自语，重复别人的话，并且喜欢背诵广告语言 | 5 | 4 | 3 | 2 | 1 |
| 11 | 表面左敝子，其实左右手都用，而且无固定使用哪只手 | 5 | 4 | 3 | 2 | 1 |
| 12 | 分不清左右方向，鞋子衣服常常穿反 | 5 | 4 | 3 | 2 | 1 |
| 13 | 对陌生地方的电梯或楼梯，不敢坐或动作缓慢 | 5 | 4 | 3 | 2 | 1 |
| 14 | 组织力不佳，经常弄乱东西，不喜欢整理自己的环境 | 5 | 4 | 3 | 2 | 1 |
| **二、** | **触觉防御** |  |  |  |  |  |
| 15 | 对亲人特别暴躁，强词夺理，到陌生环境则害怕 | 5 | 4 | 3 | 2 | 1 |
| 16 | 害怕到新场合，常常不久便要求离开 | 5 | 4 | 3 | 2 | 1 |
| 17 | 偏食、挑食，不吃青菜或软皮 | 5 | 4 | 3 | 2 | 1 |
| 18 | 害羞，不安，喜欢孤独，不爱和别人玩 | 5 | 4 | 3 | 2 | 1 |
| 19 | 容易粘妈妈或固定某人，不喜欢陌生环境，喜欢被搂抱 | 5 | 4 | 3 | 2 | 1 |
| 20 | 看电视或听故事，容易受感动，大叫或大笑，害怕恐怖镜头 | 5 | 4 | 3 | 2 | 1 |
| 21 | 严重怕黑，不喜欢在空屋，到处要人陪 | 5 | 4 | 3 | 2 | 1 |
| 22 | 早上懒床晚上睡不着，上学时常拒绝到学校，放学后又不想回家 | 5 | 4 | 3 | 2 | 1 |
| 23 | 容易生小病，生病后便不想上学，常常没有原因拒绝上学 | 5 | 4 | 3 | 2 | 1 |
| 24 | 常吸吮手指或咬指甲，不喜欢别人帮忙剪指甲 | 5 | 4 | 3 | 2 | 1 |
| 25 | 换床睡不着，不能换被子或睡衣，外出常担心睡眠问题 | 5 | 4 | 3 | 2 | 1 |
| 26 | 独占性强，别人碰他的东西，常会无缘无故发脾气 | 5 | 4 | 3 | 2 | 1 |
| 27 | 不喜欢和别人聊天，不喜欢和别人玩碰触游戏，视洗脸和洗澡为痛苦 | 5 | 4 | 3 | 2 | 1 |
| 28 | 过分保护自己的东西，尤其讨厌别人由后面接近他 | 5 | 4 | 3 | 2 | 1 |
| 29 | 怕玩沙土，有洁癖倾向 | 5 | 4 | 3 | 2 | 1 |
| 30 | 不喜欢直接视觉接触，常必须用手来表达其需要 | 5 | 4 | 3 | 2 | 1 |
| 31 | 对危险和疼痛反应迟钝或反应过于激烈 | 5 | 4 | 3 | 2 | 1 |
| 32 | 听而不见，过分安静，表情冷漠又无故嬉笑 | 5 | 4 | 3 | 2 | 1 |
| 33 | 过度安静或坚持奇怪玩法 | 5 | 4 | 3 | 2 | 1 |
| 34 | 喜欢咬人，并且常咬固定的友伴，并无故碰坏东西 | 5 | 4 | 3 | 2 | 1 |
| 35 | 内向，软弱，爱哭又常会触摸生殖器官 | 5 | 4 | 3 | 2 | 1 |
| **三、** | **本体感** |  |  |  |  |  |
| 36 | 穿脱衣裤、钮扣、拉链、系鞋带动作缓慢、笨拙 | 5 | 4 | 3 | 2 | 1 |
| 37 | 顽固，偏执，不合群，孤僻 | 5 | 4 | 3 | 2 | 1 |
| 38 | 吃饭时常掉饭粒，口水控制不住 | 5 | 4 | 3 | 2 | 1 |
| 39 | 语言不清，发音不佳，语言能力发展缓慢 | 5 | 4 | 3 | 2 | 1 |
| 40 | 懒惰，行动慢，做事没有效率 | 5 | 4 | 3 | 2 | 1 |
| 41 | 不喜欢翻跟头，打滚，爬高 | 5 | 4 | 3 | 2 | 1 |
| 42 | 上幼儿园仍不会洗手，擦脸，剪纸及自己擦屁股 | 5 | 4 | 3 | 2 | 1 |
| 43 | 上幼儿园(大，中班)仍无法用筷子，不会拿笔，攀爬或荡秋千 | 5 | 4 | 3 | 2 | 1 |
| 44 | 对小伤特别敏感，依赖他人过度照料 | 5 | 4 | 3 | 2 | 1 |
| 45 | 不善于玩积木，组合东西，排队，投球 | 5 | 4 | 3 | 2 | 1 |
| 46 | 怕爬高，拒走平衡木 | 5 | 4 | 3 | 2 | 1 |
| 47 | 到新的陌生环境很容易迷失方向 | 5 | 4 | 3 | 2 | 1 |
| **四、** | **学习能力（6岁及以上填）** |  |  |  |  |  |
| 48 | 看来有正常智慧，但学习阅读或做算数特别困难 | 5 | 4 | 3 | 2 | 1 |
| 49 | 阅读常跳字。抄写常漏宇，漏行。写字笔划常颠倒 | 5 | 4 | 3 | 2 | 1 |
| 50 | 不专心，坐不住，上课常左右看 | 5 | 4 | 3 | 2 | 1 |
| 51 | 用蜡笔着色或用笔写字写不好，写字慢而且常超出格子外 | 5 | 4 | 3 | 2 | 1 |
| 52 | 看书容易眼酸，特别害怕数学 | 5 | 4 | 3 | 2 | 1 |
| 53 | 认字能力虽好，却不知其意义，而且无法组成较长的语句 | 5 | 4 | 3 | 2 | 1 |
| 54 | 混淆背景中的特殊圆形，不易看出或认出 | 5 | 4 | 3 | 2 | 1 |
| 55 | 对老师的要求及作业无法有效完成，常有严重挫折感 | 5 | 4 | 3 | 2 | 1 |
| **五、** | **大年龄儿童特殊问题（10岁及以上填）** |  |  |  |  |  |
| 56 | 使用工具能力差，劳作或家事均做不好 | 5 | 4 | 3 | 2 | 1 |
| 57 | 自己的桌子或周围无法保持干净，收拾上很困难 | 5 | 4 | 3 | 2 | 1 |
| 58 | 对事情反应过强，无法控制情绪，容易消极 | 5 | 4 | 3 | 2 | 1 |
