# Supplementary material for: Psychometric evaluation of the Chinese revised Sensory Integration and Praxis Tests in children with amblyopia
Source: PeerJ. 2026 Jun 18;14:e21431. doi: 10.7717/peerj.21431 (PMC13283362; doi:10.7717/peerj.21431)
Supplement: Supplemental Information 2 — The analyses that pertain specifically to participants aged ¿9 years and to the SIPT-R “Specific Issues” subscale. These analyses were omitted from the main article (which is restricted to children aged 4-9 years) and are provided here for transparency and completeness. All methods and statistical procedures used below follow those described in the main text. [file peerj-14-21431-s002.docx]

**Supplementary Text 2:**

**Analyses of participants aged >9 years and the “Specific Issues” subscale**

**Manuscript:** Psychometric validation of the Chinese Revised Version of the Sensory Integration and Praxis Tests in Children with Amblyopia

**Authors:** Meng Ru^1, 2*^, Lu Pan^2, 3*^, Yuxing Huang^2^, Wuqiang Luo^2^, Lili Li^2^, Yan Luo^2^, Enwei Lin^2^, Min Kong^2^, Qi Chen^2^, Yali Luo^4^, Hairun Liu^5^, Siyan Huang^5^, Jie Li^6^, Jin Zeng^7^, Yihong Xie^1#^, Xin Xiao^2, 8, 9#^

^1^School of Public Health, Guangxi Medical University, Nanning 530021, Guangxi, China

^2^Visual Science and Optometry Center, the People's Hospital of Guangxi Zhuang Autonomous Region, Nanning 530021, Guangxi, China

^3^School of Public Health, Guilin Medical University, Guilin 541199, Guangxi, China

^4^School of Public Health and Management, Guangxi University of Chinese Medicine, Nanning 530021, Guangxi, China

^5^Cognitive Sleep Center, the People's Hospital of Guangxi Zhuang Autonomous Region, Nanning 530021, Guangxi, China

^6^Department of Children's Rehabilitation Therapy, People's Hospital of Guangxi Zhuang Autonomous Region, Nanning 530021, Guangxi, China

^7^Department of Ophthalmology, Guangdong Provincial People's Hospital (Guangdong Academy of Medical Sciences), Southern Medical University, Guangzhou 510000, Guangdong, China

^8^Department of Scientific Research, the People's Hospital of Guangxi Zhuang Autonomous Region, Nanning 530021, Guangxi, China

^9^Guangxi Key Laboratory of Eye Health, the People's Hospital of Guangxi Zhuang Autonomous Region, Nanning 530021, Guangxi, China

^*^Meng Ru and Lu Pan contributed equally to this study and should be considered co-first authors

**Corresponding Author**:

Xiao Xin^2, 8, 9^

Visual Science and Optometry Center, the People's Hospital of Guangxi Zhuang Autonomous Region, No. 6 Taoyuan Road, Nanning, Guangxi, 530021, China

Email address: [xiaoxi3891@163.com;](mailto:xiaoxi3891@163.com);)

Xie Yihong^1^

School of Public Health, Guangxi Medical University, No. 22 Shuangyong Road, Nanning, Guangxi, 530021, China

Email address: [gxxieyihong@163.com](mailto:gxxieyihong@163.com)

^#^Xiao Xin and Xie Yihong contributed equally to this work and are co-correspondence authors.

**Purpose of this supplementary file**

This supplementary file contains the analyses that pertain specifically to participants aged >9 years and to the SIPT-R “Specific Issues” subscale. These analyses were omitted from the main manuscript (which is restricted to children aged 4-9 years) and are provided here for transparency and completeness. All methods and statistical procedures used below follow those described in the main text.

**Methods**

**Participants:** analyses include all study participants aged >9 years (N = 39). Inclusion and exclusion criteria are the same as described in the main Methods section.

**Measures:** the SIPT-R questionnaire and its four subscales (Vestibular Function, Tactile Defensiveness, Proprioceptive Dysfunction and Learning Inefficiency) were scored as in the main manuscript. The “Specific Issues” subscale (designed for older children) is included in these analyses.

**Statistical analysis:** descriptive statistics are presented for demographics, clinical characteristics, and SIPT-R subscale medians (Table S1). Internal consistency was assessed using Cronbach’s α for the total scale and each subscale; split-half reliability (Spearman-Brown) is reported. Spearman rank correlations were used to examine associations between subscales and between each subscale and the total SIPT-R score. All significance tests and confidence intervals follow the conventions used in the main manuscript. Analyses were performed using the same software package as in the main manuscript. Due to the limited sample size in the >9 years group, CFA was conducted only for the <6 and 6-9 years groups.

**Results**

**Participant Demographics**

A total of 668 amblyopic children were included: 275 (41.2%) were aged ,<6 years, 354 (53.0%) were aged 6-9 years and 39 (5.8%) were aged >9 years (Table 1). Most were of Han ethnicity, and the majority had no maternal smoking or alcohol use history. A family history of myopia was more common than amblyopia in both groups. Most children had normal birth weights (2500-4000 g). The median BCVA in the worse eye was 0.4 (P_25_, P_75_: 0.3, 0.5) in the <6 years group, 0.3 (0.2, 0.4). in the 6-9 years group, and 0.3(0.2, 0.5) in the >9 years group. Moderate cases was more frequent in younger groups. Ametropic amblyopia was the predominant type (Table S1).

**Table S1 Demographics, clinical characteristics and SIPT-R Scores for Participants**

| Variable | <6 years  (N=275) | 6-9 years  (N=354) | >9 years  (N=39) |
| --- | --- | --- | --- |
| Gender (N (%)) |  |  |  |
| Boy | 143(52.0) | 181(51.1) | 22(56.4) |
| Girl | 132(48.0) | 173(48.9) | 17(43.6) |
| Ethnicity |  |  |  |
| Han | 184(66.9) | 229(64.7) | 22(56.4) |
| Ethnic minorities | 91(33.1) | 125(35.3) | 17(43.6) |
| Maternal smoking history (N (%)) |  |  |  |
| Yes | 5(1.8) | 6(1.7) | 1(2.6) |
| No | 270(98.2) | 348(98.0) | 38(97.4) |
| Maternal alcohol use history (N (%)) |  |  |  |
| Yes | 26(9.5) | 30(8.5) | 3(7.7) |
| No | 249(90.5) | 324(91.2) | 36(92.3) |
| Family history of myopia (N (%)) |  |  |  |
| Yes | 63(22.9) | 61(17.2) | 1(2.6) |
| No | 212(77.1) | 293(82.5) | 38(97.4) |
| Family history of amblyopia (N (%)) |  |  |  |
| Yes | 18(6.5) | 25(7.1) | 2(5.1) |
| No | 257(93.5) | 329(92.7) | 37(94.9) |
| Child's birth weight ((N (%)) |  |  |  |
| ≤ 2500g | 34(12.4) | 54(15.3) | 6(15.4) |
| 2500-4000g | 227(82.5) | 283(79.7) | 32(82.1) |
| ≥ 4000g | 14(5.1) | 17(4.8) | 1(2.6) |
| BCVA (Median (P_25_, P_75_)) |  |  |  |
| Better eye | 0.2(0.2, 0.4) | 0.1(0, 0.2) | 0(0, 0.2) |
| Worse eye | 0.4(0.3, 0.5) | 0.3(0.2, 0.4) | 0.3(0.2, 0.5) |
| Severity of amblyopia (N (%)) |  |  |  |
| Mild | 46(16.7) | 156(44.1) | 15(38.5) |
| Moderate | 200(72.7) | 173(48.9) | 20(51.3) |
| Severe | 29(10.5) | 25(7.1) | 4(10.3) |
| Types of amblyopia (N (%)) |  |  |  |
| Ametropic amblyopia | 167(60.7) | 183(51.7) | 13(33.3) |
| Anisometropic amblyopia | 97(35.3) | 147(41.5) | 22(56.4) |
| Strabismic amblyopia | 11(4.0) | 24(6.8) | 4(10.3) |

**Item analysis**

Across all age groups, correlations between each subscale and the total SIPT-C score were ranged from 0.773 to 0.926 (P < 0.01), indicating strong internal consistency. The inter-subscale correlations ranged from 0.482 to 0.780 (P < 0.01) (Table S2).

**Table S2 Spearman correlations of the inter-subscale and SIPT-R scores**

| **Subscales** | | 1 | 2 | 3 | 4 | 5 | 6 |
| --- | --- | --- | --- | --- | --- | --- | --- |
| **<6 years** | |  |  |  |  |  |  |
| 1 | SIPT-R | 1 |  |  |  |  |  |
| 2 | Vestibular Function | 0.835** | 1 |  |  |  |  |
| 3 | Tactile Defensiveness | 0.892** | 0.625** | 1 |  |  |  |
| 4 | Proprioceptive | 0.870** | 0.597** | 0.737** | 1 |  |  |
| **6-9 years** | |  |  |  |  |  |  |
| 1 | SIPT-R | 1 |  |  |  |  |  |
| 2 | Vestibular Function | 0.847** | 1 |  |  |  |  |
| 3 | Tactile Defensiveness | 0.916** | 0.698** | 1 |  |  |  |
| 4 | Proprioceptive | 0.851** | 0.597** | 0.780** | 1 |  |  |
| 5 | Learning Ability | 0.840** | 0.654** | 0.674** | 0.751** | 1 |  |
| **>9 years** | |  |  |  |  |  |  |
| 1 | SIPT-R | 1 |  |  |  |  |  |
| 2 | Vestibular Function | 0.773** | 1 |  |  |  |  |
| 3 | Tactile Defensiveness | 0.926** | 0.729** | 1 |  |  |  |
| 4 | Proprioceptive | 0.855** | 0.513** | 0.726** | 1 |  |  |
| 5 | Learning Ability | 0.841** | 0.482** | 0.706** | 0.714** | 1 |  |
| 6 | Specific Issues | 0.786** | 0.487** | 0.686** | 0.751** | 0.700** | 1 |

***P*<0.01

**Reliability**

The Cronbach’s α coefficients for the total scale and each subscale ranged from 0.866 to 0.969 across all age groups. Values in the <6 years group (0.911–0.969) were slightly higher than those in the 6-9 years group (0.906–0.968) and >9 years group (0.819-0.958). All split-half reliability coefficients exceeded 0.80, indicating good reliability and high internal consistency of the questionnaire (Table S3).

**Table S3 Reliability of the SIPT-R**

| Subscales | SIPT-R | Vestibular Function | Tactile Defensi-veness | Proprioc-eptive | Learning Ability | specific issues |
| --- | --- | --- | --- | --- | --- | --- |
| Items | 47/55/58 | 14 | 21 | 12 | 8 | 3 |
| <6 years |  |  |  |  |  |  |
| Cronbach’s α | 0.969 | 0.911 | 0.959 | 0.959 | **-** | **-** |
| Spearman-Brown | 0.856 | 0.883 | 0.914 | 0.954 | **-** | **-** |
| 6-9 years |  |  |  |  |  |  |
| Cronbach’s α | 0.968 | 0.906 | 0.943 | 0.930 | 0.944 | **-** |
| Spearman-Brown | 0.813 | 0.876 | 0.888 | 0.940 | 0.913 | **-** |
| >9 years |  |  |  |  |  |  |
| Cronbach’s α | 0.958 | 0.866 | 0.929 | 0.890 | 0.907 | 0.819 |
| Spearman-Brown | 0.893 | 0.826 | 0.869 | 0.830 | 0.893 | 0.883 |
