# Supplementary material for: Psychometric evaluation of the Chinese revised Sensory Integration and Praxis Tests in children with amblyopia
Source: PeerJ. 2026 Jun 18;14:e21431. doi: 10.7717/peerj.21431 (PMC13283362; doi:10.7717/peerj.21431)
Supplement: Supplemental Information 3 — The lookup rules used to transform raw scores into standardized scores across different age groups. [file peerj-14-21431-s003.docx]

**Supplementary Text 3:**

**Table for converting raw scores to standard T-score**

| **standard T-score** | **Raw score (age 3)** | | | **Raw score (age 4)** | | | | **Raw score (age 5)** | | |
| --- | --- | --- | --- | --- | --- | --- | --- | --- | --- | --- |
|  | **Vestibular Function** | **Tactile Defensiveness** | **Proprioceptive Function** | **Vestibular Function** | **Tactile Defensiveness** | **Proprioceptive Function** | **Vestibular Function** | | **Tactile Defensiveness** | **Proprioceptive Function** |
| 10 | 29 | 44 | 23 | 27 | 45 | 26 | 29 | | 50 | 24 |
| 11 | 29 | 45 | 24 | 28 | 45 | 27 | 30 | | 51 | 25 |
| 12 | 30 | 46 | 24 | 29 | 46 | 28 | 30 | | 52 | 26 |
| 13 | 30 | 47 | 25 | 29 | 47 | 28 | 31 | | 52 | 27 |
| 14 | 31 | 48 | 26 | 30 | 48 | 29 | 32 | | 53 | 27 |
| 15 | 32 | 49 | 27 | 31 | 49 | 29 | 32 | | 54 | 28 |
| 16 | 33 | 50 | 27 | 31 | 50 | 30 | 33 | | 55 | 28 |
| 17 | 33 | 51 | 28 | 32 | 51 | 30 | 34 | | 56 | 29 |
| 18 | 34 | 52 | 28 | 32 | 52 | 31 | 34 | | 56 | 29 |
| 19 | 34 | 53 | 29 | 33 | 53 | 31 | 35 | | 57 | 30 |
| 20 | 35 | 54 | 30 | 33 | 54 | 32 | 36 | | 58 | 31 |
| 21 | 36 | 55 | 30 | 34 | 55 | 32 | 36 | | 59 | 32 |
| 22 | 36 | 56 | 31 | 34 | 56 | 33 | 37 | | 60 | 33 |
| 23 | 37 | 57 | 32 | 35 | 57 | 33 | 38 | | 61 | 33 |
| 24 | 37 | 58 | 33 | 36 | 58 | 34 | 39 | | 62 | 34 |
| 25 | 38 | 59 | 33 | 36 | 59 | 34 | 39 | | 63 | 34 |
| 26 | 39 | 60 | 34 | 37 | 60 | 35 | 40 | | 63 | 35 |
| 27 | 39 | 61 | 35 | 37 | 61 | 35 | 40 | | 64 | 36 |
| 28 | 40 | 62 | 35 | 38 | 62 | 36 | 41 | | 65 | 37 |
| 29 | 40 | 63 | 36 | 39 | 63 | 36 | 42 | | 65 | 37 |
| 30 | 41 | 64 | 37 | 39 | 64 | 37 | 42 | | 67 | 38 |
| 31 | 42 | 65 | 37 | 40 | 65 | 38 | 43 | | 67 | 38 |
| 32 | 42 | 66 | 38 | 40 | 66 | 39 | 43 | | 68 | 39 |
| 33 | 43 | 67 | 38 | 41 | 67 | 39 | 44 | | 69 | 39 |
| 34 | 43 | 68 | 39 | 42 | 68 | 40 | 44 | | 70 | 40 |
| 35 | 44 | 69 | 40 | 43 | 69 | 41 | 45 | | 71 | 41 |
| 36 | 44 | 70 | 41 | 43 | 69 | 41 | 46 | | 72 | 42 |
| 37 | 45 | 71 | 41 | 44 | 70 | 42 | 46 | | 73 | 43 |
| 38 | 46 | 72 | 42 | 45 | 71 | 42 | 47 | | 73 | 43 |
| 39 | 46 | 73 | 43 | 45 | 72 | 43 | 47 | | 74 | 44 |
| 40 | 47 | 74 | 43 | 46 | 73 | 43 | 48 | | 74 | 44 |
| 41 | 47 | 75 | 44 | 47 | 74 | 44 | 49 | | 75 | 45 |
| 42 | 48 | 76 | 44 | 48 | 75 | 44 | 49 | | 76 | 45 |
| 43 | 49 | 77 | 45 | 48 | 76 | 45 | 50 | | 77 | 46 |
| 44 | 50 | 78 | 46 | 49 | 77 | 46 | 51 | | 78 | 47 |
| 45 | 50 | 79 | 46 | 49 | 78 | 46 | 51 | | 79 | 47 |
| 46 | 50 | 80 | 47 | 49 | 78 | 47 | 52 | | 80 | 48 |
| 47 | 51 | 82 | 47 | 50 | 79 | 47 | 53 | | 81 | 48 |
| 48 | 52 | 83 | 48 | 51 | 80 | 48 | 53 | | 82 | 49 |
| 49 | 52 | 84 | 49 | 52 | 80 | 49 | 54 | | 83 | 49 |
| 50 | 53 | 85 | 50 | 52 | 81 | 49 | 54 | | 84 | 50 |
| 51 | 54 | 86 | 50 | 53 | 82 | 50 | 55 | | 85 | 51 |
| 52 | 54 | 87 | 51 | 53 | 83 | 51 | 56 | | 86 | 52 |
| 53 | 55 | 88 | 52 | 54 | 84 | 51 | 57 | | 87 | 53 |
| 54 | 55 | 89 | 53 | 54 | 85 | 52 | 57 | | 88 | 53 |
| 55 | 56 | 90 | 53 | 55 | 86 | 52 | 58 | | 89 | 54 |
| 56 | 56 | 91 | 54 | 55 | 87 | 53 | 58 | | 89 | 54 |
| 57 | 57 | 92 | 54 | 56 | 88 | 53 | 59 | | 90 | 55 |
| 58 | 57 | 93 | 55 | 57 | 89 | 54 | 59 | | 91 | 56 |
| 59 | 58 | 94 | 56 | 57 | 90 | 54 | 60 | | 92 | 56 |
| 60 | 59 | 95 | 56 | 58 | 91 | 55 | 61 | | 93 | 57 |
| 61 | 59 | 96 | 57 | 59 | 92 | 56 | 62 | | 93 | 57 |
| 62 | 60 | 97 | 58 | 59 | 93 | 56 | 62 | | 94 | 58 |
| 63 | 61 | 98 | 58 | 60 | 94 | 57 | 63 | | 95 | 59 |
| 64 | 62 | 99 | 59 | 60 | 95 | 57 | 64 | | 96 | 59 |
| 65 | 62 | 100 | 60 | 61 | 96 | 58 | 64 | | 97 | 60 |
| 66 | 63 | 101 | 60 | 62 | 97 | 58 | 65 | | 97 |  |
| 67 | 63 | 102 |  | 63 | 97 | 59 | 65 | | 98 |  |
| 68 | 64 | 103 |  | 64 | 98 | 60 | 66 | | 99 |  |
| 69 | 64 | 104 |  | 65 | 99 |  | 66 | | 100 |  |
| 70 | 65 | 105 |  | 65 | 100 |  | 67 | | 101 |  |

| **standard T-score** | **Raw score (age 6)** | | | | **Raw score (age 7)** | | | |
| --- | --- | --- | --- | --- | --- | --- | --- | --- |
|  | **Vestibular Function** | **Tactile Defensiveness** | **Proprioceptive Function** | **Learning Ability** | **Vestibular Function** | **Tactile Defensiveness** | **Proprioceptive Function** | **Learning Ability** |
| 10 | 30 | 51 | 31 | 10 | 31 | 52 | 27 | 11 |
| 11 | 30 | 52 | 31 | 10 | 32 | 53 | 28 | 12 |
| 12 | 31 | 53 | 32 | 11 | 33 | 54 | 29 | 12 |
| 13 | 32 | 54 | 32 | 11 | 33 | 54 | 30 | 13 |
| 14 | 33 | 55 | 33 | 12 | 34 | 55 | 30 | 13 |
| 15 | 34 | 56 | 33 | 12 | 34 | 56 | 31 | 14 |
| 16 | 34 | 57 | 34 | 13 | 35 | 57 | 32 | 14 |
| 17 | 35 | 58 | 34 | 13 | 35 | 58 | 32 | 15 |
| 18 | 35 | 58 | 35 | 14 | 36 | 59 | 33 | 15 |
| 19 | 36 | 59 | 35 | 14 | 37 | 60 | 33 | 16 |
| 20 | 37 | 60 | 36 | 15 | 38 | 61 | 34 | 16 |
| 21 | 37 | 61 | 36 | 15 | 39 | 62 | 34 | 17 |
| 22 | 38 | 62 | 37 | 16 | 40 | 63 | 35 | 17 |
| 23 | 38 | 63 | 37 | 16 | 40 | 64 | 35 | 18 |
| 24 | 39 | 64 | 38 | 17 | 41 | 64 | 36 | 18 |
| 25 | 40 | 65 | 38 | 17 | 42 | 65 | 36 | 19 |
| 26 | 40 | 66 | 39 | 18 | 42 | 66 | 37 | 19 |
| 27 | 41 | 67 | 39 | 19 | 43 | 67 | 38 | 20 |
| 28 | 42 | 68 | 40 | 20 | 44 | 68 | 39 | 21 |
| 29 | 42 | 69 | 40 | 20 | 44 | 69 | 39 | 21 |
| 30 | 43 | 70 | 41 | 21 | 45 | 70 | 40 | 22 |
| 31 | 44 | 71 | 41 | 21 | 45 | 71 | 40 | 23 |
| 32 | 45 | 72 | 42 | 21 | 46 | 72 | 41 | 23 |
| 33 | 45 | 73 | 42 | 22 | 47 | 73 | 42 | 23 |
| 34 | 46 | 74 | 43 | 22 | 48 | 74 | 43 | 24 |
| 35 | 47 | 75 | 43 | 23 | 8 | 75 | 43 | 24 |
| 36 | 48 | 76 | 44 | 23 | 49 | 76 | 44 | 25 |
| 37 | 49 | 78 | 44 | 24 | 50 | 77 | 44 | 25 |
| 38 | 49 | 79 | 45 | 25 | 50 | 78 | 45 | 26 |
| 39 | 50 | 80 | 45 | 25 | 51 | 79 | 45 | 26 |
| 40 | 50 | 81 | 46 | 26 | 51 | 80 | 46 | 27 |
| 41 | 50 | 81 | 46 | 26 | 52 | 81 | 47 | 27 |
| 42 | 51 | 82 | 47 | 27 | 53 | 82 | 48 | 28 |
| 43 | 52 | 83 | 47 | 27 | 54 | 83 | 48 | 28 |
| 44 | 53 | 84 | 48 | 28 | 54 | 84 | 49 | 29 |
| 45 | 54 | 85 | 49 | 28 | 55 | 85 | 49 | 29 |
| 46 | 55 | 86 | 50 | 29 | 55 | 85 | 50 | 30 |
| 47 | 55 | 87 | 50 | 29 | 56 | 86 | 51 | 30 |
| 48 | 56 | 88 | 51 | 30 | 57 | 87 | 52 | 31 |
| 49 | 56 | 88 | 51 | 30 | 57 | 88 | 52 | 31 |
| 50 | 57 | 89 | 52 | 31 | 58 | 89 | 53 | 32 |
| 51 | 58 | 90 | 52 | 32 | 58 | 90 | 53 | 32 |
| 52 | 59 | 90 | 53 | 32 | 59 | 91 | 54 | 33 |
| 53 | 59 | 91 | 53 | 33 | 60 | 92 | 54 | 33 |
| 54 | 60 | 92 | 54 | 33 | 61 | 93 | 55 | 34 |
| 55 | 60 | 93 | 54 | 34 | 61 | 93 | 55 | 34 |
| 56 | 61 | 94 | 55 | 34 | 62 | 94 | 56 | 35 |
| 57 | 62 | 95 | 55 | 35 | 63 | 95 | 56 | 35 |
| 58 | 62 | 96 | 56 | 35 | 64 | 96 | 57 | 36 |
| 59 | 63 | 97 | 57 | 36 | 65 | 97 | 57 | 36 |
| 60 | 64 | 98 | 57 | 36 | 65 | 98 | 58 | 37 |
| 61 | 64 | 99 | 57 | 37 | 66 | 99 | 58 | 37 |
| 62 | 65 | 100 | 58 | 38 | 66 | 100 | 59 | 38 |
| 63 | 65 | 101 | 58 | 38 | 67 | 101 | 59 | 38 |
| 64 | 66 | 102 | 59 | 39 | 68 | 102 | 60 | 39 |
| 65 | 67 | 103 | 59 | 39 | 68 | 103 |  | 40 |
| 66 | 68 | 104 | 60 | 40 | 69 | 104 |  |  |
| 67 | 69 | 105 |  |  | 69 | 105 |  |  |
| 68 | 69 |  |  |  | 70 |  |  |  |
| 69 | 70 |  |  |  |  |  |  |  |
| 70 |  |  |  |  |  |  |  |  |

| **standard T-score** | **Raw score (age 8)** | | | | | **Raw score (age 9)** | | | |
| --- | --- | --- | --- | --- | --- | --- | --- | --- | --- |
|  | **Vestibular Function** | **Tactile Defensive-ness** | **Proprioce-ptive Function** | **Learning Ability** | **Vestibular Function** | | **Tactile Defensive-ness** | **Proprioce-ptive Function** | **Learning Ability** |
| 10 | 31 | 48 | 21 | 9 | 26 | | 45 | 23 | 10 |
| 11 | 31 | 49 | 22 | 10 | 27 | | 46 | 24 | 11 |
| 12 | 32 | 50 | 22 | 10 | 28 | | 47 | 25 | 11 |
| 13 | 33 | 51 | 23 | 11 | 29 | | 49 | 26 | 12 |
| 14 | 34 | 52 | 23 | 11 | 29 | | 50 | 27 | 12 |
| 15 | 34 | 53 | 24 | 12 | 30 | | 51 | 27 | 13 |
| 16 | 35 | 54 | 25 | 12 | 31 | | 52 | 28 | 13 |
| 17 | 35 | 54 | 26 | 13 | 32 | | 53 | 28 | 14 |
| 18 | 36 | 55 | 27 | 14 | 33 | | 54 | 29 | 14 |
| 19 | 37 | 56 | 28 | 14 | 33 | | 55 | 30 | 15 |
| 20 | 38 | 57 | 28 | 15 | 34 | | 56 | 31 | 16 |
| 21 | 38 | 58 | 29 | 16 | 35 | | 57 | 32 | 16 |
| 22 | 39 | 59 | 30 | 16 | 36 | | 58 | 32 | 17 |
| 23 | 39 | 60 | 31 | 17 | 37 | | 59 | 33 | 17 |
| 24 | 40 | 61 | 32 | 17 | 38 | | 60 | 34 | 18 |
| 25 | 40 | 62 | 32 | 18 | 38 | | 61 | 34 | 18 |
| 26 | 41 | 63 | 33 | 18 | 39 | | 62 | 35 | 19 |
| 27 | 42 | 64 | 34 | 19 | 40 | | 63 | 35 | 19 |
| 28 | 42 | 65 | 34 | 19 | 41 | | 64 | 36 | 20 |
| 29 | 43 | 66 | 35 | 20 | 42 | | 65 | 37 | 21 |
| 30 | 44 | 67 | 36 | 20 | 43 | | 66 | 37 | 21 |
| 31 | 45 | 68 | 36 | 21 | 43 | | 67 | 38 | 22 |
| 32 | 45 | 69 | 37 | 21 | 44 | | 68 | 39 | 22 |
| 33 | 46 | 70 | 38 | 22 | 44 | | 70 | 39 | 23 |
| 34 | 46 | 71 | 39 | 23 | 45 | | 71 | 40 | 23 |
| 35 | 47 | 72 | 39 | 23 | 45 | | 72 | 41 | 24 |
| 36 | 48 | 73 | 40 | 24 | 46 | | 73 | 42 | 24 |
| 37 | 49 | 74 | 41 | 25 | 47 | | 74 | 42 | 25 |
| 38 | 50 | 75 | 41 | 25 | 48 | | 75 | 43 | 25 |
| 39 | 50 | 76 | 42 | 26 | 49 | | 76 | 44 | 26 |
| 40 | 51 | 77 | 43 | 26 | 50 | | 77 | 44 | 26 |
| 41 | 52 | 78 | 44 | 27 | 51 | | 78 | 45 | 27 |
| 42 | 53 | 79 | 44 | 27 | 52 | | 79 | 46 | 27 |
| 43 | 53 | 80 | 45 | 28 | 53 | | 80 | 46 | 28 |
| 44 | 54 | 81 | 46 | 29 | 53 | | 81 | 47 | 28 |
| 45 | 54 | 82 | 46 | 29 | 54 | | 82 | 48 | 29 |
| 46 | 55 | 83 | 47 | 30 | 55 | | 83 | 48 | 30 |
| 47 | 56 | 84 | 48 | 30 | 55 | | 84 | 49 | 31 |
| 48 | 57 | 85 | 49 | 31 | 56 | | 85 | 50 | 31 |
| 49 | 57 | 86 | 50 | 31 | 57 | | 86 | 51 | 32 |
| 50 | 58 | 87 | 51 | 32 | 57 | | 87 | 51 | 32 |
| 51 | 58 | 88 | 52 | 33 | 58 | | 88 | 52 | 33 |
| 52 | 59 | 89 | 52 | 33 | 59 | | 89 | 52 | 33 |
| 53 | 60 | 90 | 53 | 34 | 60 | | 91 | 53 | 34 |
| 54 | 60 | 91 | 54 | 35 | 61 | | 92 | 54 | 34 |
| 55 | 61 | 92 | 55 | 35 | 62 | | 93 | 54 | 35 |
| 56 | 62 | 93 | 56 | 36 | 63 | | 94 | 55 | 36 |
| 57 | 63 | 94 | 57 | 37 | 63 | | 95 | 56 | 36 |
| 58 | 64 | 95 | 57 | 37 | 64 | | 96 | 56 | 37 |
| 59 | 64 | 96 | 58 | 38 | 65 | | 97 | 57 | 37 |
| 60 | 65 | 97 | 58 | 38 | 65 | | 98 | 57 | 38 |
| 61 | 65 | 98 | 59 | 39 | 66 | | 99 | 58 | 38 |
| 62 | 66 | 99 | 59 | 39 | 67 | | 100 | 59 | 39 |
| 63 | 67 | 100 | 60 | 40 | 67 | | 101 | 60 | 39 |
| 64 | 67 | 101 |  |  | 68 | | 102 |  | 40 |
| 65 | 68 | 102 |  |  | 69 | | 103 |  |  |
| 66 | 69 | 103 |  |  | 70 | | 104 |  |  |
| 67 | 69 | 104 |  |  |  | | 105 |  |  |
| 68 | 70 | 105 |  |  |  | |  |  |  |
| 69 |  |  |  |  |  | |  |  |  |
| 70 |  |  |  |  |  | |  |  |  |

| **standard T-score** | **Raw score (age 10)** | | | | | **Raw score (age 11)** | | | |
| --- | --- | --- | --- | --- | --- | --- | --- | --- | --- |
|  | **Vestibular Function** | **Tactile Defensive-ness** | **Proprioce-ptive Function** | **Learning Ability** | **Vestibular Function** | | **Tactile Defensive-ness** | **Proprioce-ptive Function** | **Learning Ability** |
| 10 | 31 | 49 | 26 | 8 | 30 | | 47 | 27 | 13 |
| 11 | 32 | 50 | 27 | 8 | 31 | | 48 | 28 | 13 |
| 12 | 33 | 51 | 27 | 8 | 31 | | 49 | 28 | 14 |
| 13 | 33 | 52 | 28 | 9 | 32 | | 50 | 29 | 15 |
| 14 | 34 | 53 | 28 | 9 | 33 | | 51 | 30 | 15 |
| 15 | 34 | 54 | 29 | 9 | 33 | | 52 | 31 | 16 |
| 16 | 35 | 55 | 29 | 9 | 34 | | 54 | 31 | 16 |
| 17 | 35 | 56 | 30 | 10 | 35 | | 55 | 32 | 17 |
| 18 | 36 | 57 | 31 | 11 | 35 | | 56 | 32 | 17 |
| 19 | 37 | 58 | 31 | 11 | 36 | | 57 | 33 | 18 |
| 20 | 38 | 59 | 32 | 12 | 37 | | 58 | 34 | 18 |
| 21 | 38 | 60 | 33 | 12 | 38 | | 59 | 34 | 19 |
| 22 | 39 | 61 | 34 | 13 | 38 | | 60 | 35 | 19 |
| 23 | 40 | 62 | 34 | 14 | 39 | | 61 | 36 | 20 |
| 24 | 41 | 63 | 35 | 14 | 40 | | 62 | 36 | 20 |
| 25 | 41 | 63 | 36 | 15 | 41 | | 63 | 37 | 21 |
| 26 | 42 | 64 | 36 | 16 | 42 | | 64 | 38 | 21 |
| 27 | 43 | 65 | 37 | 16 | 42 | | 65 | 38 | 22 |
| 28 | 44 | 66 | 37 | 17 | 43 | | 66 | 39 | 22 |
| 29 | 44 | 67 | 38 | 18 | 43 | | 67 | 39 | 23 |
| 30 | 45 | 68 | 39 | 18 | 44 | | 68 | 40 | 23 |
| 31 | 46 | 69 | 39 | 19 | 45 | | 69 | 41 | 24 |
| 32 | 46 | 70 | 40 | 19 | 46 | | 71 | 42 | 24 |
| 33 | 47 | 71 | 41 | 20 | 47 | | 72 | 42 | 25 |
| 34 | 48 | 72 | 41 | 21 | 47 | | 73 | 43 | 25 |
| 35 | 48 | 73 | 42 | 21 | 48 | | 74 | 44 | 26 |
| 36 | 49 | 74 | 43 | 22 | 49 | | 75 | 44 | 26 |
| 37 | 50 | 75 | 43 | 22 | 50 | | 76 | 45 | 27 |
| 38 | 51 | 76 | 44 | 23 | 51 | | 77 | 45 | 27 |
| 39 | 52 | 77 | 44 | 24 | 52 | | 78 | 46 | 28 |
| 40 | 52 | 78 | 45 | 24 | 52 | | 79 | 47 | 28 |
| 41 | 53 | 79 | 46 | 25 | 53 | | 80 | 47 | 29 |
| 42 | 53 | 80 | 47 | 26 | 54 | | 81 | 48 | 29 |
| 43 | 54 | 80 | 47 | 26 | 55 | | 82 | 48 | 30 |
| 44 | 55 | 81 | 48 | 27 | 56 | | 83 | 49 | 31 |
| 45 | 55 | 82 | 48 | 28 | 57 | | 84 | 49 | 31 |
| 46 | 56 | 83 | 49 | 28 | 57 | | 85 | 50 | 32 |
| 47 | 57 | 84 | 49 | 29 | 58 | | 86 | 51 | 32 |
| 48 | 57 | 85 | 50 | 30 | 58 | | 87 | 51 | 33 |
| 49 | 58 | 86 | 51 | 31 | 59 | | 88 | 52 | 33 |
| 50 | 59 | 87 | 51 | 31 | 60 | | 89 | 52 | 34 |
| 51 | 59 | 88 | 52 | 32 | 61 | | 91 | 53 | 34 |
| 52 | 60 | 89 | 52 | 32 | 62 | | 92 | 54 | 35 |
| 53 | 61 | 90 | 53 | 33 | 62 | | 93 | 54 | 35 |
| 54 | 62 | 91 | 54 | 33 | 63 | | 94 | 55 | 36 |
| 55 | 62 | 92 | 54 | 34 | 63 | | 95 | 56 | 36 |
| 56 | 63 | 93 | 55 | 35 | 64 | | 96 | 56 | 37 |
| 57 | 64 | 94 | 56 | 36 | 65 | | 97 | 57 | 37 |
| 58 | 64 | 95 | 56 | 36 | 65 | | 98 | 58 | 38 |
| 59 | 65 | 96 | 57 | 37 | 66 | | 99 | 58 | 38 |
| 60 | 66 | 97 | 57 | 37 | 67 | | 100 | 59 | 39 |
| 61 | 67 | 98 | 58 | 38 | 67 | | 101 | 59 | 39 |
| 62 | 67 | 99 | 59 | 39 | 68 | | 102 | 60 | 40 |
| 63 | 68 | 100 | 59 | 39 | 68 | | 103 |  |  |
| 64 | 69 | 101 | 60 | 40 | 69 | | 104 |  |  |
| 65 | 70 | 102 |  |  | 70 | | 105 |  |  |
| 66 |  | 103 |  |  |  | |  |  |  |
| 67 |  | 104 |  |  |  | |  |  |  |
| 68 |  | 105 |  |  |  | |  |  |  |
| 69 |  |  |  |  |  | |  |  |  |
| 70 |  |  |  |  |  | |  |  |  |

| **standard T-score** | **Raw score (age 12)** | | | |
| --- | --- | --- | --- | --- |
|  | **Vestibular Function** | **Tactile Defensiveness** | **Proprioceptive Function** | **Learning Ability** |
| 10 | 37 | 51 | 27 | 12 |
| 11 | 37 | 51 | 28 | 13 |
| 12 | 38 | 52 | 28 | 13 |
| 13 | 38 | 53 | 29 | 14 |
| 14 | 39 | 54 | 30 | 14 |
| 15 | 40 | 55 | 31 | 15 |
| 16 | 41 | 56 | 31 | 16 |
| 17 | 41 | 57 | 32 | 16 |
| 18 | 42 | 58 | 33 | 17 |
| 19 | 42 | 59 | 33 | 17 |
| 20 | 43 | 60 | 34 | 18 |
| 21 | 44 | 61 | 34 | 18 |
| 22 | 44 | 63 | 35 | 19 |
| 23 | 45 | 64 | 36 | 19 |
| 24 | 45 | 65 | 36 | 20 |
| 25 | 46 | 66 | 37 | 20 |
| 26 | 46 | 67 | 38 | 21 |
| 27 | 47 | 68 | 38 | 21 |
| 28 | 47 | 69 | 39 | 22 |
| 29 | 48 | 70 | 39 | 22 |
| 30 | 49 | 71 | 40 | 23 |
| 31 | 49 | 72 | 41 | 23 |
| 32 | 50 | 73 | 41 | 24 |
| 33 | 51 | 74 | 42 | 24 |
| 34 | 51 | 75 | 43 | 25 |
| 35 | 52 | 76 | 43 | 25 |
| 36 | 52 | 77 | 44 | 26 |
| 37 | 53 | 78 | 44 | 26 |
| 38 | 54 | 79 | 45 | 27 |
| 39 | 54 | 80 | 46 | 27 |
| 40 | 55 | 81 | 46 | 28 |
| 41 | 56 | 82 | 47 | 28 |
| 42 | 56 | 83 | 48 | 29 |
| 43 | 57 | 84 | 48 | 29 |
| 44 | 58 | 85 | 49 | 30 |
| 45 | 58 | 86 | 49 | 31 |
| 46 | 59 | 87 | 50 | 31 |
| 47 | 59 | 88 | 51 | 32 |
| 48 | 60 | 89 | 51 | 32 |
| 49 | 61 | 90 | 52 | 33 |
| 50 | 61 | 91 | 52 | 33 |
| 51 | 62 | 92 | 53 | 34 |
| 52 | 62 | 93 | 54 | 34 |
| 53 | 63 | 94 | 54 | 35 |
| 54 | 63 | 95 | 55 | 36 |
| 55 | 64 | 96 | 56 | 36 |
| 56 | 54 | 97 | 56 | 37 |
| 57 | 65 | 98 | 57 | 37 |
| 58 | 66 | 99 | 58 | 38 |
| 59 | 66 | 100 | 58 | 38 |
| 60 | 67 | 101 | 59 | 39 |
| 61 | 67 | 102 | 59 | 39 |
| 62 | 68 | 103 | 60 | 40 |
| 63 | 68 | 104 |  |  |
| 64 | 69 | 105 |  |  |
| 65 | 69 |  |  |  |
| 66 | 70 |  |  |  |
| 67 |  |  |  |  |
| 68 |  |  |  |  |
| 69 |  |  |  |  |
| 70 |  |  |  |  |
