# Supplementary material for: Psychometric evaluation of the Chinese revised Sensory Integration and Praxis Tests in children with amblyopia
Source: PeerJ. 2026 Jun 18;14:e21431. doi: 10.7717/peerj.21431 (PMC13283362; doi:10.7717/peerj.21431)

**Supplementary Text 4：**

**English-Chinese translation of the reference**

Chinese Mental Health Journal, 1994, Vol. 8, No. 4, pp. 145-147

**Preliminary Report on application of Child Sensory Integration Scale**

Ren Guiying, Wang Yufeng, Gu Bomei, Shen Jianlin

Institute of Mental Health, Beijing Medical University

**Abstract:**

Child Sensory Integration Scale, developed by a Taiwan psychologist, was first introduced to mainland China and administered to 1994 school children in urban Beijing urban area. With good acceptance and easeiness to understand, all the items were kept unchanged in application. The test-retest and split-half reliability for the scale were 0.47-0.73 and 0.68-0.77, respectively. Factor analysis identified 6 principal factors accounting for 80.7% variance, which was slightly different from the report of original author. The implications and limitations of application of this scale was discussed.

**Keywords:** Child Sensory Integration Scale, sensory integration, reliability, validity

The theory of Sensory Integration was first proposed by the American clinical psychologist Ayres A.J. (1972). She pointed out that sensory integration involves combining the sensory information input from various parts of the human body, processed by the brain, to complete the response to internal and external perceptions of the body. Only through sensory integration can different parts of the nervous system work together in a coordinated manner, enabling smooth interaction between the individual and the environment [1]. When this system cannot function normally, it is termed "Sensory Integration Dysfunction"[1-3]. Ayres believed that sensory integration dysfunction includes body movement disorders, structural and spatial perception disorders, vestibular balance dysfunction, auditory-language disorders, and tactile defensive disorders (Clark F.A., 1985) [4].

To research sensory integration, Ayres provided a checklist for each type of sensory integration dysfunction to be completed by parents [1-2]. The scale includes situations from the past six months and earlier. Based on the Chinese cultural background, Taiwan's Fan Boxiong (1985) merged several comprehensive symptom checklists to compile a Sensory Integration Checklist and tested it on elementary school students from first to sixth grade in Taiwan. The test homogeneity coefficient was 0.93; the split-half correlation coefficients were 0.41-0.99; test-retest reliability was 0.47-0.82. The correlation between the checklist and the Quick Neurological Screening Test (Mutti et al., 1978) was 0.52 (P < 0.01); the correlation with the Southern California Sensory Integration Tests (Ayres, 1980) was 0.70-0.95. This indicates that the reliability and validity of the checklist are satisfactory [5]. The purpose of this study is to understand the usability and acceptability of the "Sensory Integration Rating Scale" in Mainland China. The test results of the scale are now reported as follows.

**Materials and Methods**

**Research Subjects** A total of 1994 intellectually normal students from grades 1-6 at four representative ordinary primary schools in Beijing, including 963 boys and 1031 girls (male:female ratio 1:1.07).

**Research Methods**

Research Tool:

Sensory Integration Rating Scale: The Sensory Integration Checklist compiled by Taiwan's Zheng Xinxiong was used. This checklist is a synthesis of Ayres' checklists for sensory integration dysfunction symptoms. It consists of 58 questions. Rated on a five-point scale of "Never, Seldom, Sometimes, Often, Always", with "Never" scored the highest and "Always" scored the lowest. The scale has five items:

(1) Gross Muscle and Balance: Mainly involves the body's gross motor abilities. Includes 14 questions such as "Clumsy hands and feet, prone to falling."

(2) Tactile Over-Defensiveness and Emotional Instability: Mainly involves emotional stability and excessive behaviors. Includes 21 questions such as "Shy, uneasy, doesn't like to play with others."

(3) Poor Proprioception; Poor Body Coordination: Mainly involves the body's sense of proprioception and balance coordination abilities. Includes 12 questions such as "Slow in putting on/taking off clothes, tying shoelaces."

(4) Underdeveloped Learning Abilities or Poor Coordination: Mainly involves insufficient learning abilities caused by poor sensory integration. Includes 8 questions such as "Often misses words when reading, often misses words or lines when copying, writing strokes often reversed."

1. Special Problems for Older Ages: This involves 3 questions related to using tools and doing household chores. Only assessed for children aged 10 and above. The full score for each item of the scale is 100 points, a score below 60 indicates a problem.

Assessment Method:

The examiners trained the homeroom teachers to ensure unified understanding. Then, the homeroom teachers distributed the questionnaires to the parents, explained them to the parents, and the parents filled them out independently. Out of 2228 enrolled students, 2035 valid questionnaires were returned, a recovery rate of 91.3%. This study excluded 41 children with intellectual abnormalities (IQ < 70), the remaining 1994 were the subjects of this study. Among them, 208 students were retested with the Sensory Integration Rating Scale after 3-5 weeks, a retest rate of 10.4%.

Statistical Methods:

The collected questionnaire data were entered into a Facom M-340 computer, and the ANALYST statistical software package was used for statistical analysis.

**Results**

**General Information** The subjects of this study were 1994 individuals. The distribution of numbers in each age group is shown in Table 1.

**Table 1. Distribution of Numbers in Each Age Group**

| Age group | Boy | Girl | Total |
| --- | --- | --- | --- |
| 6 years | 125 | 125 | 250 |
| 7 years | 148 | 145 | 293 |
| 8 years | 159 | 157 | 3316 |
| 9 years | 116 | 142 | 258 |
| 10 years | 198 | 207 | 405 |
| 11 years and above | 217 | 255 | 472 |
| Total | 963 | 1031 | 1994 |

**Reliability Analysis of the Sensory Integration Rating Scale**

Test-Retest Reliability:

208 intellectually normal students were retested with the Sensory Integration Rating Scale after 3-5 weeks. The test-retest correlation coefficients were: Gross Muscle and Balance item: r=0.67 (P<0.01); Tactile Over-Defensiveness: r=0.73 (P<0.01); Poor Proprioception: r=0.47 (P<0.01); Underdeveloped Learning Abilities: r=0.71 (P<0.01); Special Problems item: r=0.61 (P<0.01). This indicates that this rating scale has good test-retest reliability.

Split-Half Reliability:

Split-half correlation test was performed based on the odd/even numbers of the questions. Since the Special Problems item had only 3 questions, its correlation coefficient was not calculated. The split-half correlation coefficients for the other four items were respectively: Gross Muscle and Balance: r=0.72 (P<0.001); Tactile Over-Defensiveness: r=0.77 (P<0.001); Poor Proprioception: r=0.68 (P<0.001); Underdeveloped Learning Abilities: r=0.77 (P<0.01). This suggests that the internal consistency of this scale is stable.

Homogeneity Reliability:

The various items of the Sensory Integration Scale represent specific contents, but there are significant correlations between them. This suggests that the homogeneity of the scale is good (Table 2).

**Table 2 Correlations Among the Five Items of the Sensory Integration Rating Scale**

|  | Gross Muscle & Balance | Tactile Defensiveness | Proprioceptive Deficits | Learning Disability |
| --- | --- | --- | --- | --- |
| Gross Muscle & Balance | 1.00 |  |  |  |
| Tactile Defensiveness | 0.55*** | 1.00 |  |  |
| Proprioceptive Deficits | 0.49*** | 0.63*** | 1.00 |  |
| Learning Disability | 0.48*** | 0.45*** | 0.53*** | 1.00 |
| Labor & Tool Use | 0.44*** | 0.46*** | 0.51*** | 0.55*** |

***P<0.001

**Validity Analysis of the Sensory Integration Rating Scale**

Factor analysis was performed on the results of 1994 intellectually normal children, selecting six representative factors with eigenvalues greater than 1, the cumulative contribution rate was 80.7%. Items with factor loadings greater than 0.4 were selected. Factor F1 is Learning Disability, F2 is Tactile Over-Defensiveness, F3 is Vestibular Balance and Proprioceptive Deficits, F4 is Emotional Instability, F5 is Hyperactive Behavior, F6 is Vestibular Stimulation Input. As shown in Table 3 and Table 4: The structural validity of the Sensory Integration Rating Scale we used is good.

Correlations with the Four Items of the Sensory Integration Rating Scale: From the results in Table 4, it can be seen that F1 factor (Learning Disability), F2 factor (Sensory Over-Defensiveness), F4 factor (Emotional Instability), and F3 factor (Vestibular Balance and Proprioceptive Deficits) all have high correlations (0.80-0.94) with the content represented by the original scale. F5 factor (Hyperactive Behavior) has relatively high correlations with the Gross Muscle & Balance and Learning Disability items (0.70, 0.64). F6 factor (Vestibular Stimulation Input) only has a moderate correlation with the Gross Muscle & Balance item.

**Table 3 Factor Analysis Results of the Sensory Integration Rating Scale for 1994 Intellectually Normal Children**

| Factor 1 | | Factor 2 | | | Factor 3 | | Factor 4 | | Factor 5 | | | Factor 6 | |
| --- | --- | --- | --- | --- | --- | --- | --- | --- | --- | --- | --- | --- | --- |
| Item | Loading | Item | Loading | Item | | Loading | Item | Loading | Item | Loading | Item | | Loading |
| 48 | 0.6980 | 33 | 0.5454 | 36 | | 0.5383 | 15 | 0.5148 | 8 | 0.6355 | 1 | | 0.7446 |
| 52 | 0.6979 | 28 | 0.5029 | 42 | | 0.5081 | 16 | 0.5095 | 7 | 0.6008 | 2 | | 0.6498 |
| 49 | 0.6280 | 32 | 0.4891 | 43 | | 0.5081 | 19 | 0.5092 | 9 | 0.5662 |  | |  |
| 55 | 0.6124 | 18 | 0.4637 | 46 | | 0.4634 | 21 | 0.4806 | 50 | 0.4063 |  | |  |
| 50 | 0.5787 | 37 | 0.4595 | 5 | | 0.4163 | 20 | 0.4575 |  |  |  | |  |
| 54 | 0.5648 | 40 | 0.4540 |  | |  |  |  |  |  |  | |  |
| 51 | 0.5629 | 29 | 0.4332 |  | |  |  |  |  |  |  | |  |
| 52 | 0.4953 | 34 | 0.4267 |  | |  |  |  |  |  |  | |  |
| 57 | 0.4233 | 27 | 0.4044 |  | |  |  |  |  |  |  | |  |
| 40 | 0.4163 | 26 | 0.5029 |  | |  |  |  |  |  |  | |  |

**Table 4. Correlations Between Factors and the Four Items of the Sensory Integration Rating Scale**

|  | Factor 1 | Factor 2 | Factor 3 | Factor 4 | Factor 5 | Factor 6 |
| --- | --- | --- | --- | --- | --- | --- |
| Gross Muscle & Balance | 0.52*** | 0.41*** | 0.47*** | 0.47*** | 0.70*** | 0.49*** |
| Tactile Defensiveness | 0.49*** | 0.85*** | 0.49*** | 0.80*** | 0.41*** | 0.09* |
| Proprioceptive Deficits | 0.58*** | 0.59*** | 0.85*** | 0.47*** | 0.35*** | 0.05* |
| Learning Disability | 0.94*** | 0.37*** | 0.41*** | 0.34*** | 0.64*** | 0.08** |

*P<0.05, **P<0.01, ***P<0.001

**Evaluation of the Scale's Application**

The Sensory Integration Scale has been applied in both Eastern and Western cultural contexts [1,5]. Application results show that the Sensory Integration Scale has good reliability and validity. After introducing the Sensory Integration Scale, we conducted tests: test-retest reliability 0.47-0.73; split-half reliability 0.68-0.77; homogeneity reliability 0.44-0.63, once again confirming that this scale has good reliability. The good correlation (0.49-0.94) between the factors derived from scale structural validity and factor analysis and the original items also confirms the scale's good validity. Additionally, the issues reflected by the scale's items are phenomena that occur in children's daily lives. The expression is simple, clear, and easy for parents to answer, indicating the scale's acceptability is relatively good. The scale's items have broad coverage and are not very difficult; parents or caregivers with a medium level of education can generally complete it in about 20 minutes. This indicates the scale's usability is also very good.

Based on our testing situation and research results on sensory integration therapy[6], this scale can be used to assess the development of children's sensory integration abilities and the severity of sensory integration dysfunction, and can also serve as a tool for comparing therapeutic effects before and after sensory integration therapy.

**References**

Ottenbacher Kenneth, Short Margaret A. ; Sensory integrative dysfunction in children; A review of theory and treatment. Advance in Development and Behavior Pediatrics, 1985: 6: 287—329

--Liao Wenshu. Children's Sensory Integration, Psychological Publishing House. Taiwan, 1991.

--Zheng Boxiong. How to Help Children with Learning Difficulties, Yuanliu Publishing Enterprise Co., Ltd. Taiwan, 1992 (8th edition printing).

Robert A. Cummins; Sensory integration and learning disabilities; Ayres'factor analyses reappraised, J. Learning Disabilities, 1991, 24: 160—168.

--Zheng Boxiong. Sensory Integration Checklist Norms and Manual for Shrinking and Scaling Test (internal exchange material).

--Ren Guiying et al. Clinical efficacy observation of sensory integration therapy methods (to be published).

Lorna Jean King. A sensory integrative approach to schizophrenic, geriatric and autistic population, Sensory Integrative Therapy and Research, 1990: 7: 1—110 (Japanese).


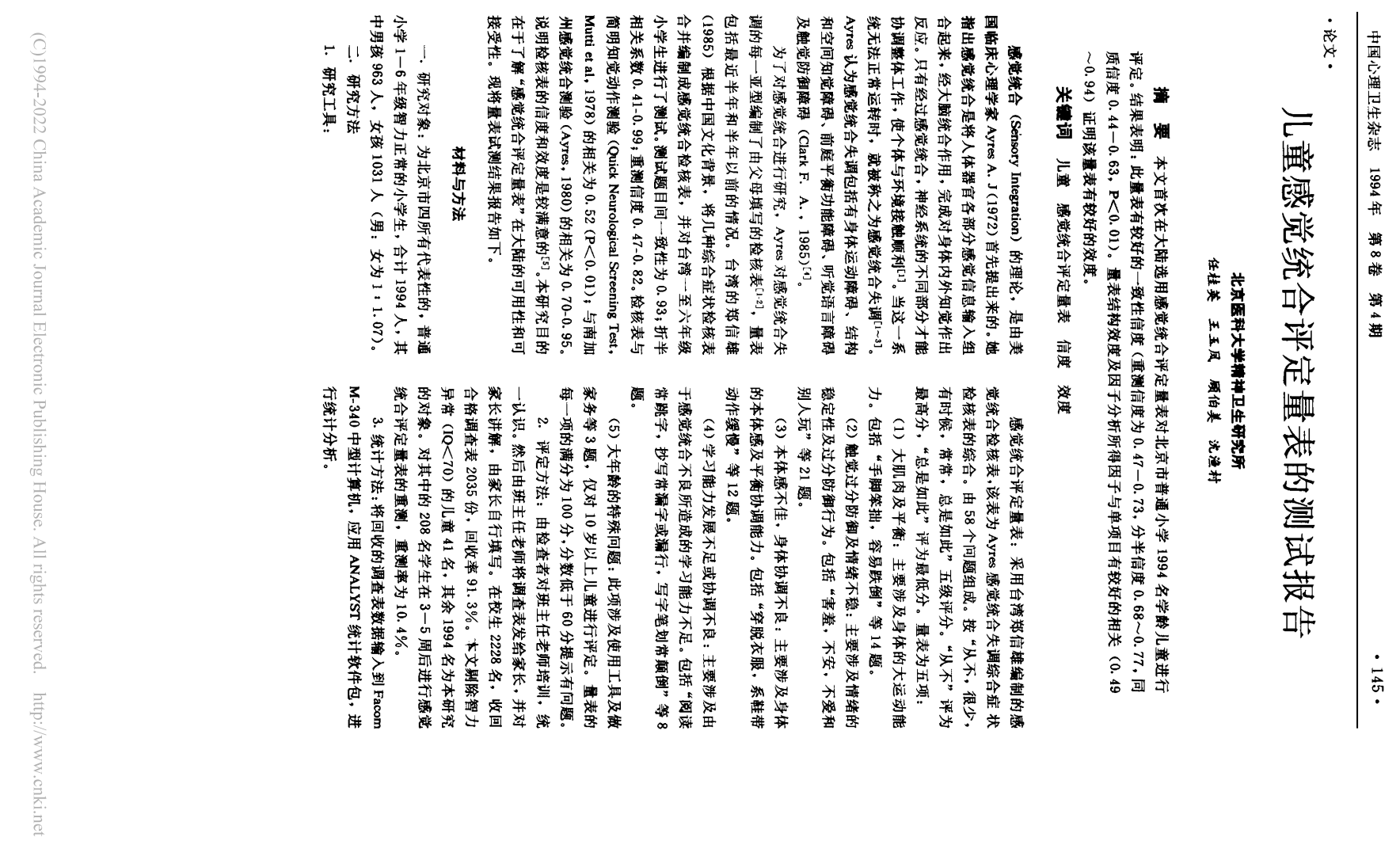

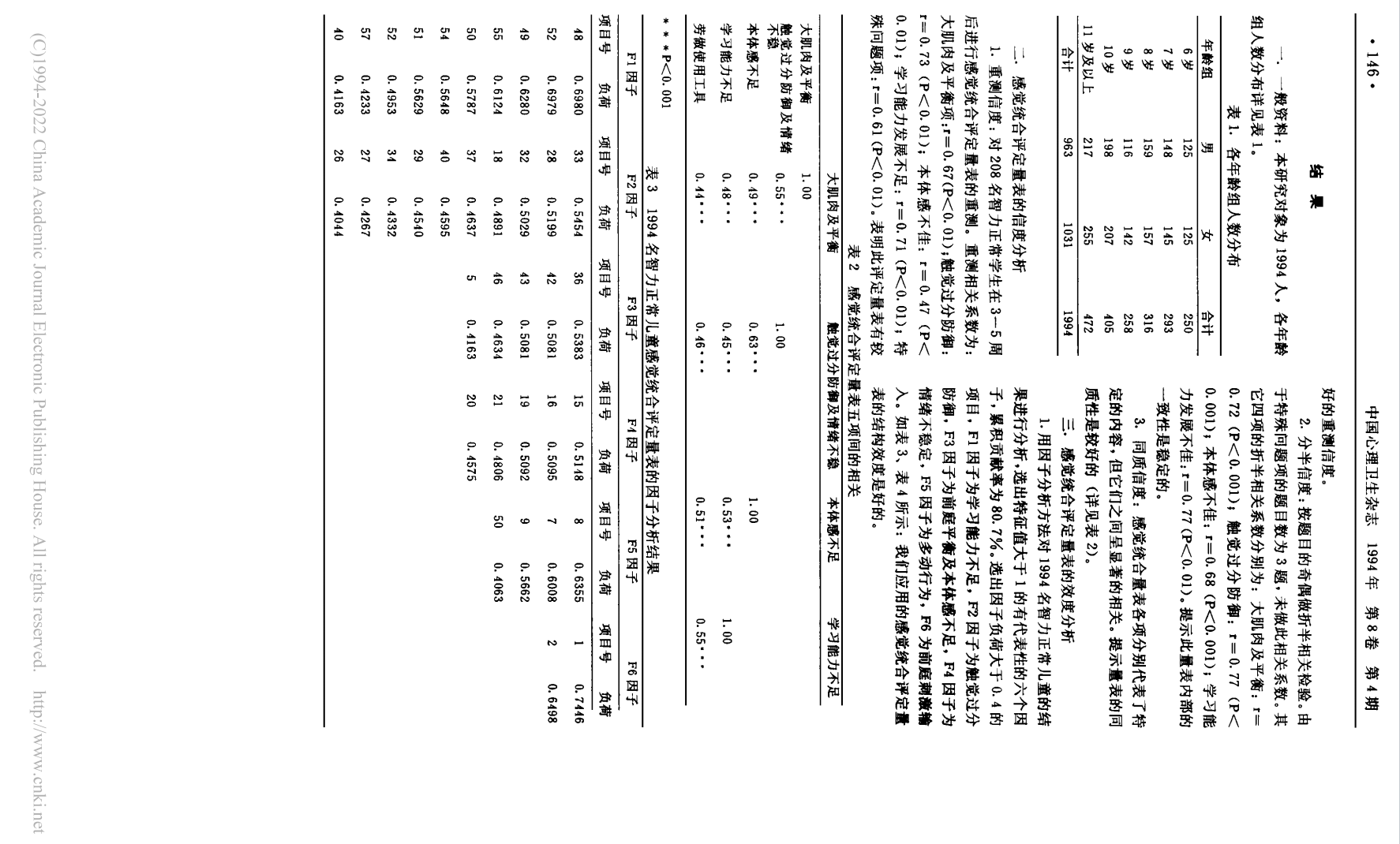

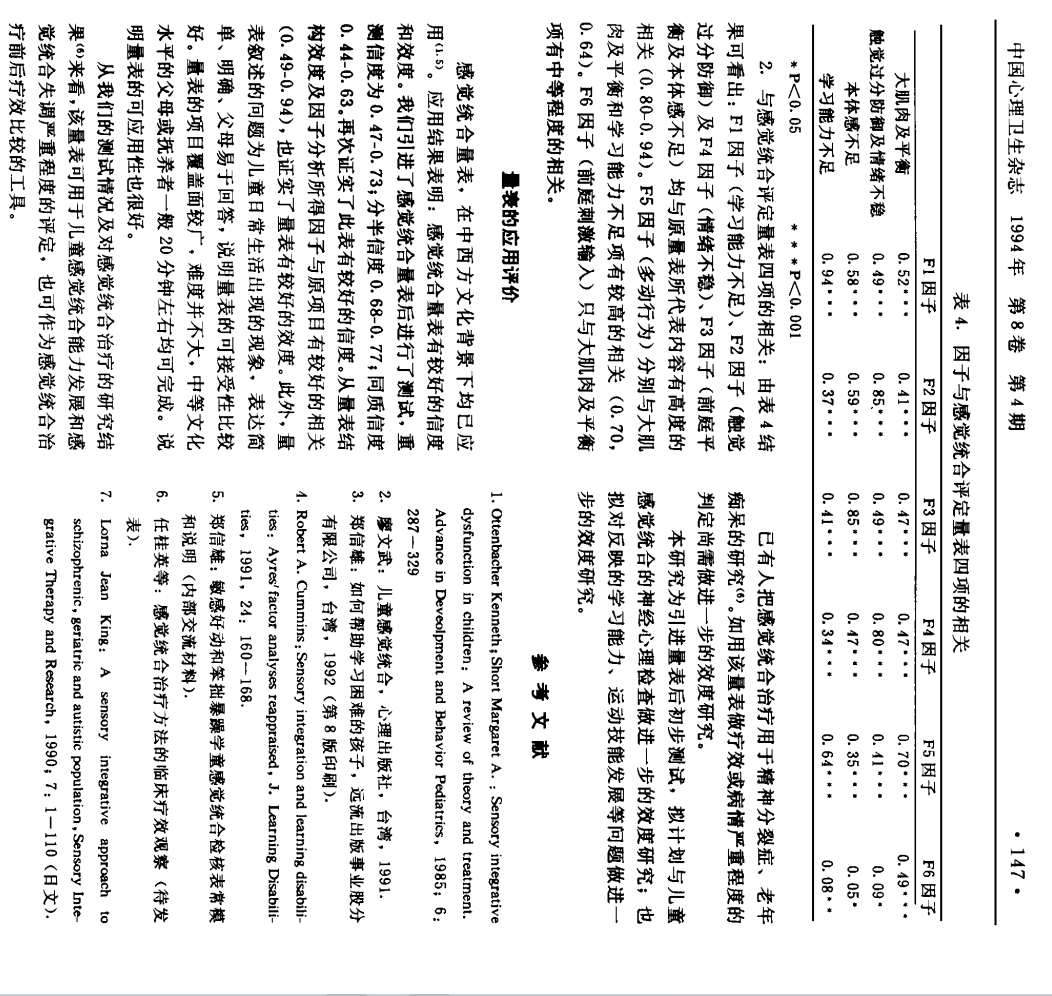

Supplement: Supplemental Information 4 [file peerj-14-21431-s004.docx]
