# Supplementary material for: Psychometric evaluation of the Chinese revised Sensory Integration and Praxis Tests in children with amblyopia
Source: PeerJ. 2026 Jun 18;14:e21431. doi: 10.7717/peerj.21431 (PMC13283362; doi:10.7717/peerj.21431)
Supplement: Supplemental Information 5 — Each entry describes a variable name, its meaning, coding values, and scoring direction to facilitate interpretation and reuse of the dataset. [file peerj-14-21431-s005.docx]

**Supplementary Codebook:**

**Codebook of the Raw Data**

**Manuscript:** Psychometric validation of the Chinese Revised Version of the Sensory Integration and Praxis Tests in Children with Amblyopia

**Authors:** Meng Ru^1, 2*^, Lu Pan^2, 3*^, Yuxing Huang^2^, Wuqiang Luo^2^, Lili Li^2^, Yan Luo^2^, Enwei Lin^2^, Min Kong^2^, Qi Chen^2^, Yali Luo^4^, Hairun Liu^5^, Siyan Huang^5^, Jie Li^6^, Jin Zeng^7^, Yihong Xie^1#^, Xin Xiao^2, 8, 9#^

^1^School of Public Health, Guangxi Medical University, Nanning 530021, Guangxi, China

^2^Visual Science and Optometry Center, the People's Hospital of Guangxi Zhuang Autonomous Region, Nanning 530021, Guangxi, China

^3^School of Public Health, Guilin Medical University, Guilin 541199, Guangxi, China

^4^School of Public Health and Management, Guangxi University of Chinese Medicine, Nanning 530021, Guangxi, China

^5^Cognitive Sleep Center, the People's Hospital of Guangxi Zhuang Autonomous Region, Nanning 530021, Guangxi, China

^6^Department of Children's Rehabilitation Therapy, People's Hospital of Guangxi Zhuang Autonomous Region, Nanning 530021, Guangxi, China

^7^Department of Ophthalmology, Guangdong Provincial People's Hospital (Guangdong Academy of Medical Sciences), Southern Medical University, Guangzhou 510000, Guangdong, China

^8^Department of Scientific Research, the People's Hospital of Guangxi Zhuang Autonomous Region, Nanning 530021, Guangxi, China

^9^Guangxi Key Laboratory of Eye Health, the People's Hospital of Guangxi Zhuang Autonomous Region, Nanning 530021, Guangxi, China

^*^Meng Ru and Lu Pan contributed equally to this study and should be considered co-first authors

**Corresponding Author**:

Xiao Xin^2, 8, 9^

Visual Science and Optometry Center, the People's Hospital of Guangxi Zhuang Autonomous Region, No. 6 Taoyuan Road, Nanning, Guangxi, 530021, China

Email address: [xiaoxi3891@163.com;](mailto:xiaoxi3891@163.com);)

Xie Yihong^1^

School of Public Health, Guangxi Medical University, No. 22 Shuangyong Road, Nanning, Guangxi, 530021, China

Email address: [gxxieyihong@163.com](mailto:gxxieyihong@163.com)

^#^Xiao Xin and Xie Yihong contributed equally to this work and are co-correspondence authors.

| **Variable name** | **Code** | **Meaning** |
| --- | --- | --- |
| Gender | 1 | Boy |
|  | 2 | Girl |
| Ethnicity | 1 | Han |
|  | 2 | Ethnic minorities |
| Types of amblyopia | 1 | Mild |
|  | 2 | Moderate |
|  | 3 | Severe |
| Severity of amblyopia | 1 | Ametropic amblyopia |
|  | 2 | Anisometropic amblyopia |
|  | 3 | Strabismic amblyopia |
| Child's birth weight | 1 | ≤ 2500g |
|  | 2 | 2500-4000g |
|  | 3 | ≥ 4000g |
| Maternal smoking history | 1 | Yes |
|  | 2 | No |
| Maternal alcohol use history | 1 | Yes |
|  | 2 | No |
| Family history of myopia | 1 | Yes |
|  | 2 | No |
| Family history of amblyopia | 1 | Yes |
|  | 2 | No |
| A1-A14 | 1=Never, 2=Rarely, 3=Sometimes, 4=Often, 5=Always | Vestibular Function items  (14 items, Likert scale) |
| B15-B35 | Same as above | Tactile Defensiveness items  (21 items, Likert scale) |
| C36-C47 | Same as above | Proprioceptive items  (12 items, Likert scale) |
| D48-D55 | Same as above | Learning Ability items  (8 items, Likert scale) |
| E56-E58 | Same as above | Specific Issues items  (3 items, Likert scale) |

| **Variable** | **Description** |
| --- | --- |
| A_before | Raw total score of Vestibular Function (sum of A1 - A14) |
| A_mean | Mean score of Vestibular Function (A_before / 14 items) |
| A_trans | Standardized T-score of Vestibular Function (raw total converted to T-score, mean = 50, SD = 10, age-normed) |
| B_before | Raw total score of Tactile Defensiveness (sum of B15 - B35) |
| B_mean | Mean score of Tactile Defensiveness (B_before / 21 items) |
| B_trans | Standardized T-score of Tactile Defensiveness |
| C_before | Raw total score of Proprioceptive Function (sum of C36 - C47) |
| C_mean | Mean score of Proprioceptive Function (C_before / 12 items) |
| C_trans | Standardized T-score of Proprioceptive Function |
| D_before | Raw total score of Learning Ability (sum of D48 - D55) |
| D_mean | Mean score of Learning Ability (D_before / 8 items) |
| D_trans | Standardized T-score of Learning Ability |
| E_before | Raw total score of Specific Issues (sum of E56 - E58) |
| E_mean | Mean score of Specific Issues (E_before / 3 items) |
| E_trans | Standardized T-score of Specific Issues |
